# Efficacy and safety of Danlou tablets in the treatment of stable angina pectoris with intermingled phlegm and blood stasis syndrome in coronary heart disease: a multicenter randomized controlled study

**DOI:** 10.3389/fcvm.2024.1462730

**Published:** 2024-10-30

**Authors:** Zeng Li, Han Li, Zheng Li, Yushi Zhou, Wei Yang, Yuhan Ao, Xinghua Xiang, Chongchai Li, Mingxue Zhang

**Affiliations:** ^1^ Liaoning University of Traditional Chinese Medicine, Shenyang, Liaoning, China; ^2^ Shenyang Health Service Center, Shenyang, Liaoning, China; ^3^ Affiliated Hospital of Liaoning University of Traditional Chinese Medicine, Shenyang, Liaoning, China; ^4^ Institute of Clinical Medicine, Basic Sciences, Chinese Academy of Traditional Chinese Medicine, Beijing, China

**Keywords:** coronary heart disease, stable angina pectoris, syndrome of mutual accumulation of phlegm and blood stasis, Danlou tablets, randomized controlled

## Abstract

**Objectives:**

In this study, we assessed the clinical efficacy and safety of Danlou tablets in the treatment of stable angina pectoris (SAP) with intermingled phlegm and blood stasis (IPBS), to provide high-quality evidence-based medical evidence for the prevention and treatment of coronary heart disease.

**Methods:**

In this multicenter randomized controlled study, 304 patients diagnosed with stable angina pectoris with IPBS enrolled from 12 national traditional Chinese medicine (TCM) clinical research centers in China were randomly assigned to the treatment group and the control group at a ratio of 1:1. Each group was divided into four subgroups based on the results of TCM syndrome differentiation: IPBS, IPBS combined with qi deficiency, IPBS combined with qi stagnation, and IPBS combined with toxin accumulation. The control group was treated with routine Western medicine. In addition to routine Western medicine treatment, the treatment group (the IPBS group) was treated with Danlou tablets or Danlou tablets supplemented by interventional therapies based on the results of traditional Chinese medicine differentiation. The frequency of angina attacks per week was the main efficacy evaluation indicator and the secondary efficacy evaluation indicators included angina symptom score, Seattle Angina Questionnaire, an electrocardiogram (ECG) efficacy evaluation, a cardiac Doppler two-dimensional ultrasound, an electrocardiogram treadmill exercise test, blood lipids, blood glucose, a coagulation function test, hemorheology indicators, homocysteine, C-reactive protein (CRP) or high sensitivity-CRP, TCM syndromes (syndrome score, tongue, pulse), and long-term prognosis (endpoint outcome, cardiovascular events).

**Results:**

There were 300 cases in the full analysis set (FAS), 266 in the per-protocol set (PPS), and 300 in the safety set. Regarding the main efficacy indicator, after treatment, the reduction in the frequency of weekly angina attacks in the treatment group was significantly greater than that in the control group (*P* < 0.05). The results of the FAS and PPS were consistent. Regarding the secondary efficacy evaluation indicators, the angina symptom, TCM syndrome, ECG evaluation, Seattle Angina Pectoris Questionnaire, and 36-item Health Status Survey Summary Form scores of the treatment group were significantly higher than the control group (*P* < 0.05) and the homocysteine levels of the treatment group were significantly reduced (*P* < 0.05). The results of the FAS and PPS were consistent. In the PPS, the triglyceride levels in the treatment group were significantly lower than those in the control group after treatment (*P* < 0.05). The activated partial thromboplastin time in the treatment group decreased significantly (*P* < 0.05). There was no statistically significant difference in the safety indicators and incidence of adverse reactions between the two groups.

**Conclusion:**

Treatment with Danlou tablets and the modified combination therapy based on Western medicine treatment could improve angina pectoris symptoms of patients with SAP and IPBS syndrome and its concurrent syndromes, and improve patients’ quality of life. Furthermore, the treatment is safe, has a long-term prognosis, and is worth further promotion and application in clinical practice.

**Clinical Trial Registration:**

https://www.chictr.org.cn/showproj.html?proj=39724, ChiCTR registry, ChiCTR1900023708.

## 1 Introduction

Coronary heart disease (CHD) is the leading cause of death worldwide, with a continuously increasing trend, and is one of the major diseases that seriously threaten human health (1). According to the 2020 China Cardiovascular Health and Disease Report, the current number of cardiovascular disease patients in China is approximately 330 million, including 11.39 million patients with coronary heart disease (2). Western medicine usually treats coronary heart disease by controlling risk factors, using secondary preventive drugs, promoting cardiac recovery, and intervening with surgical treatment if necessary, which delay the progress of the disease and reduce the incidence rate and mortality. However, there is still an urgent need to address issues such as recurrent symptoms of angina pectoris, poor patient quality of life, and repeated hospitalization (3, 4). Traditional Chinese medicine (TCM) and traditional Chinese patent medicines are multi-component, multi-target, multi-way, multi-effect, synergistic, and safe, making the role of traditional Chinese medicine in disease prevention and treatment prominent (5). However, current high-level research focuses on particular diseases and specific traditional Chinese medicine therapies, and there is a lack of high-level clinical research on major diseases. Traditional randomized controlled trials are often used, which makes it difficult to take into account the complex system of multidimensional syndrome elements in patients in reality, and also difficult to reflect the advantages of dynamic diagnosis and treatment in traditional Chinese medicine. In this context, it is imperative to adapt to the development of evidence-based traditional Chinese medicine, optimize existing diagnosis and treatment plans, and demonstrate the advantages of traditional Chinese medicine in distinguishing and treating suitable populations. Therefore, seeking a safe, effective, and improved combination of traditional Chinese and Western medicine diagnosis and treatment plan is particularly important to improve patient prognosis and quality of life.

The syndrome of intermingled phlegm and blood stasis (IPBS) (Supplementary Material S1) in coronary heart disease is characterized by multiple vessel lesions and moderate to severe stenosis, while the non-IPBS syndrome is characterized by single vessel lesions and mild stenosis. A literature review of the clinical research on coronary heart disease in the past 40 years shows that the proportion of heart blood stasis obstruction and phlegm turbidity internal obstruction is gradually increasing (6). In clinical practice, the syndrome of IPBS often does not exist independently and often coexists with other syndrome elements. Epidemiological investigations and studies on a large sample of syndromes have shown that phlegm, blood stasis, qi stagnation (QS), toxic pathogens, and qi deficiency (QD) are the main syndrome elements of stable angina pectoris in coronary heart disease. Danlou tablets are the only listed traditional Chinese patent medicine and simple preparation recommended for the treatment of IPBS syndrome with stable angina pectoris in the Chinese Medicine Diagnosis and Treatment Guide for Stable Angina Pectoris (evidence level: B; recommended intensity: conditional recommendation) and is composed of Gualou, Xiebai, Gegen, Chuanxiong, Danshen, Chishao, Zexie, Huangqi, Gusuibu, and Yujin. Danlou tablets are effective in widening the chest and promoting yang qi circulation, reducing phlegm and resolving masses, and promoting blood circulation and resolving blood stasis, which has a significant clinical effect in treating stable angina pectoris caused by IPBS (7, 8). Gao’s meta-analysis study showed that Danlou tablets have good effects by improving the cessation rate of nitroglycerin in patients with coronary heart disease, reducing the frequency of angina attacks, and shortening the duration of angina attacks (9). Multiple basic studies have shown that Danlou tablets can reduce serum resistin levels, inhibit the activation of the ERK pathway, effectively suppress smooth muscle cell proliferation, and inhibit the secretion of ET and the expression of NO, thereby protecting vascular endothelial cells; increasing the expression of PPAR-γ and LXR; reducing total tholesterol (TC), triglyceride (TG), and low-density lipoprotein cholesterol (LDL-C) levels; increasing high-density lipoprotein cholesterol (HDL-C); and improving lipid metabolism disorders to delay the occurrence and development of coronary heart disease (10, 11). Therefore, in this study, we considered the characteristics of individualized syndrome differentiation and treatment in traditional Chinese medicine clinical practice, divided the research population based on their combination of disease and syndrome, and selected different accompanying syndrome elements under the same disease and syndrome type to distinguish between and evaluate the population. Patients with stable angina pectoris with IPBS were treated with Danlou tablets and its modified treatment according to the their main symptoms, secondary symptoms, furred tongue, pulse, and other factors. Thus a large-sample multicenter randomized controlled study based on the main disease and main syndrome intervention plan combined with evidence-based intervention measures was conducted to explore the diagnostic and therapeutic advantages of an individualized syndrome differentiation and treatment plan of Western medicine combined with traditional Chinese medicine, as well as the clinical efficacy and safety of Danlou tablets, to provide high-level evidence-based medical evidence for the clinical treatment of coronary heart disease.

## 2 Methods

### 2.1 Ethical approval

This study was approved by the ethics committee of the Affiliated Hospital of Liaoning University of Traditional Chinese Medicine [No. 2019003FS (KT)-003-02] and other co-participating centers, and was performed in accordance with the ethical standards as laid down in the 1964 Declaration of Helsinki and its later amendments or comparable ethical standards. All participants in the study signed the informed consent forms.

### 2.2 Study design

This was a multicenter randomized controlled trial conducted at 12 national TCM clinical research centers and was registered at the China Clinical Trial Registration Center (ChiCTR registry number, ChiCTR1900023708).

### 2.3 Patients

The 304 patients enrolled in this study were from 12 national TCM clinical research centers in six major regions of China between June 2019 and August 2020. The 12 national TCM clinical research centers included Liaoning University of Traditional Chinese Medicine Affiliated Hospital, Beijing University of Traditional Chinese Medicine Dongzhimen Hospital, Shaanxi University of Traditional Chinese Medicine Affiliated Hospital, Guangxi University of Traditional Chinese Medicine First Affiliated Hospital, Yunnan Provincial Hospital of Traditional Chinese Medicine, Southwest Medical University Affiliated Hospital of Traditional Chinese Medicine, Shanghai University of Traditional Chinese Medicine Affiliated Longhua Hospital, Guangzhou University of Traditional Chinese Medicine First Affiliated Hospital, Hubei Provincial Hospital of Traditional Chinese Medicine, Gansu Provincial Hospital of Traditional Chinese Medicine, Chengdu University of Traditional Chinese Medicine Affiliated Hospital, and The First Affiliated Hospital of Heilongjiang University of Traditional Chinese Medicine. The ethics committees of each research center approved and supervised the research protocol.

The patients strictly met the diagnostic criteria of IPBS or related concurrent syndromes in stable angina of coronary heart disease. These patients were aged between 35 and 75 years old and had not taken any traditional Chinese medicine within the previous 2 weeks. All the patients voluntarily participated in the clinical observation, cooperated with follow-up, and signed informed consent forms. Patients with the following conditions were excluded:

(1) Disease exclusion criteria:
  (a) Heart failure patients with valvular heart disease, various types of cardiomyopathy, malignant arrhythmia, and NYHA heart function grading III–IV;
  (b) Patients with untreated or uncontrolled hypertension (BP ≥ 180/110 mmHg);
  (c) Patients with severe cerebrovascular disease;
  (d) Patients with severe pulmonary insufficiency (PaO_2_ < 60 mmHg), serious primary diseases of the endocrine and hematopoietic system, moderate and severe liver insufficiency (aminotransferase level is three times higher than the upper limit of normal value) or moderate and severe renal insufficiency (eGFR < 60 ml/min/1.73 m^2^) or new acute cerebrovascular diseases in the previous 3 months;
  (e) Patients with psychosis;
  (f) Pregnant or lactating patients;
  (g) Patients who had participated in other clinical trials within the previous 3 months;
  (h) Patients with a life expectancy of less than 1 year;
  (i) Patients with an allergic constitution or allergic to traditional Chinese medicine;
  (j) Patients who were unwilling or unable to receive clinical follow-up.
(2) Excluded patients with symptoms that did not match the efficacy and main treatment of the intervention drug Danlou tablets (Yin deficiency syndrome, heat syndrome), and excluded those with concurrent symptoms that Danlou tablets could solve (Yang deficiency syndrome, cold syndrome).

### 2.4 Diagnostic criteria

For the traditional Chinese medicine diagnostic criteria for IPBS and its concurrent syndrome, we referred to the 2008 “Guidelines for the Diagnosis and Treatment of Common Diseases in Traditional Chinese Medicine (Part of Western Medicine Diseases),” “Clinical Diagnosis and Treatment Terminology of Traditional Chinese Medicine—Symptoms Part,” “Guiding Principles for Clinical Research of New Chinese Medicines (2002 Edition),” and “Diagnosis and Treatment Effectiveness Standards for Traditional Chinese Medicine Diseases (2012 Edition),” combined with expert argumentation. Patients with the following primary symptoms and two or more secondary symptoms, combined with tongue symptoms and pulse, were diagnosed with IPBS:

(a) Primary symptoms: Chest tightness and chest or precordial pain, characterized by pricking pain, tightness, or severe pain;
(b) Secondary symptoms: heavy head and body fatigue, nausea, vomiting or generalized vomiting, obese body with excessive phlegm, and cyan, light purple, or dark purple complexion, lips, nails, or skin;
(c) Tongue and pulse: purple tongue (light purple, dark purple, or cyan purple), ecchymosis or petechiae, greasy or slippery fur on tongue, or astringent or slippery pulse string.

The diagnosis of the concurrent syndromes had the following characteristics based on the syndrome of IPBS. IPBS-QD: recurrent mild chest pain, chest tightness, shortness of breath, palpitations, easy to sweat, fatigue, slurred speech, pale complexion, slightly dark tongue or with tooth marks, thin white fur on tongue, weak pulse, or slow and intermittent pulse; IPBS-QS: distention and pain in the chest, back, and lateral thorax, epigastric stuffiness, and stringy pulse; IPBS-TA: asthma accompanied by wheezing or suffocation, red spots or brocade patterns on the face, body rash, purple (or dark) tongue, crimson tongue, dull and withered tongue, mealy tongue fur, prickly (red, white, or black spots) tongue, or a swollen tongue.

For the diagnostic criteria of stable angina pectoris, we referred to the 2007 China Guidelines for the Diagnosis and Treatment of Chronic Stable Angina Pectoris, the 2013 ESC Guidelines for the Management of Stable Coronary Heart Disease (12), the updated guidelines for the diagnosis and management of stable ischemic heart disease patients jointly released by ACC/AHA/AATS/PCNA/SCAI/STS in 2014 (13), and the expert consensus on the non-invasive imaging pathways for stable coronary heart disease from 2017. The patients were defined as having clinical symptoms of stable angina pectoris if they met any of the following criteria:

(a) Significant ST-T dynamic changes in an electrocardiogram (ECG) during the onset of chest pain, or resting ECG with ST-segment depression or T-wave inversion, but presenting as “pseudo normalization” during the onset of chest pain; or a 24 h dynamic ECG showing ST-T changes consistent with symptoms;
(b) For patients with resting ECG abnormalities, left bundle branch block (LBBB), ST-segment decline >1 mm, pacing rhythm, pre-excitation syndrome, and other ECG exercise tests that were difficult to accurately evaluate, and a positive load function test (echocardiography, nuclide myocardial perfusion imaging, and cardiac magnetic resonance imaging);
(c) A positive exercise test according to the Bruce protocol;
(d) Coronary CT or coronary angiography showing at least one main branch or branch stenosis >50%.

### 2.5 Randomization

The patients were first stratified according to the national TCM clinical research center, and the central randomized system generated a static regional random sequence. The patients diagnosed with stable angina pectoris with IPBS syndrome were entered into the corresponding group at a ratio of 1:1 according to the assigned random sequence. Centralized random sequence distribution was adopted to achieve the principle of distribution concealment. The administrator of the random center did not participate in any part of the implementation of the trial or follow-up. The outcome assessors were blinded. Due to the treatment nature, the participants and physicians could not be masked. The patients were assigned to subgroups before randomization with distribution concealment and the outcome assessors were blinded so as to minimize selection bias to the greatest extent possible.

### 2.6 Interventions and treatment courses

The patients in the control group received routine Western medicine treatment [antiplatelet drugs, lipid-lowering drugs, β receptor blockers, nitrates or long-acting calcium antagonists, angiotensin-converting enzyme inhibitor (ACEI)/angiotensin receptor blocker (ARB)]. The treatment group received a combination of modified Danlou tablets and routine Western medicine treatment as follows:

(a) For IPBS: Danlou tablets (produced by Jilin KangNaiEr Company, batch number, 20181109, 0.3 g × 15 tablets, 5 tablets at a time, 3 times per day) should be administered;
(b) If the syndrome elements of qi deficiency, qi stagnation, and toxin accumulation were combined with IPBS, one main syndrome element should be selected, and the following interventions [granules, equivalent to the dosage of decoction pieces (produced by Jiangyin Tianjiang Pharmaceutical Company)] should be added on the basis of IPBS treatment: IPBS-QD: 15 g of *Codonopsis pilosula*, 15 g of *Poria cocos*, and 9 g of Banxia (*Pinellia ternata*) should be added; IPBS-QS: 10 g of *Rhizoma corydalis*, 10 g of *Fructus aurantii immaturus*, and 9 g of *P. ternata* should be added; IPBS-TA: 10 g of *Rheum officinale*, 15 g of *Polygonum cuspidatum*, and 9 g of *P. ternata* should be added. Each variety of concomitant granules should be administered one bag at a time, dissolved in 100 ml of boiling water at 90–100℃, and taken warm, three times a day.

Treatment courses: all patients received 24 weeks of treatment.

### 2.7 Outcomes and assessments

The main efficacy evaluation indicator was the average weekly frequency of angina attacks. Secondary efficacy evaluation indicators included angina score, Seattle Angina Questionnaire, an ECG efficacy evaluation, a cardiac Doppler two-dimensional ultrasound, an electrocardiogram treadmill exercise test (exercise test was conducted only after the doctor had evaluated the safety and obtained informed consent from the patient), blood lipids, blood glucose, coagulation function test, hemorheology indicators, high sensitivity C-reactive protein (hs-CRP, some sub-centers detected CRP due to laboratory restrictions), homocysteine (Hcy), TCM syndrome score, and the Health Status Survey Summary Form (SF-36). Safety indicators included blood routine, liver function, kidney function, and urine/stool routine. The endpoint outcome indicators included cardiogenic death; non-fatal myocardial infarction; successful cardiac arrest after resuscitation; in need of revascularization surgery; and cardiovascular events such as stroke, unstable angina pectoris, or hospitalization for heart failure. The efficacy evaluation indicators were measured at the 0th month (1 week ± 7 days), 1st month (4 weeks ± 7 days), 3rd month (12 weeks ± 7 days), and 6th month (24 weeks ± 7 days) after patient enrollment, and one telephone follow-up was conducted in the 3rd month (36 weeks ± 7 days) and 6th month (48 weeks ± 7 days) after the end of treatment to collect the endpoint outcomes and adverse events data.

The criteria for the angina symptom score were as follows: Significant effect, a reduction of ≥70% in angina symptom score; effective, angina symptom score reduction of ≥30% and <70%; ineffective, angina symptom score reduced by <30%; aggravated: angina symptom score reduced by <0%. The criteria for determining the efficacy of traditional Chinese medicine syndrome integration are as follows: Significant effect, a significant improvement in clinical symptoms and signs, with a reduction of ≥70% in syndrome integration; effective, clinical symptoms and signs had improved, with a reduction of ≥30% in syndrome integration; ineffective, clinical symptoms and signs had not significantly improved, or even worsened, with a reduction of <30% in syndrome integration; aggravated, clinical symptoms and signs had worsened, with a reduction of <0 in syndrome integration. The efficacy evaluation criteria for the ECGs were as follows: Significant effect, the ECG returned to “approximately normal” (i.e., “normal range”) or reached “normal electrocardiogram”; effective, the depressed ST segment rebounded above 0.05 mV after treatment but did not reach normal levels, the inverted T-wave changed in the main leads became shallower (up to 25% or more)or T-wave changed from flat to upright, with improvement in atrioventricular or ventricular conduction block; ineffective, the ECG was approximately the same as before treatment; Aggravated: the ST segment decreased by more than 0.05 mV compared to before treatment, with a deepening of inverted T waves in the main lead (up to 25%), flattening of upright T waves, inversion of flat T waves, and the occurrence of ectopic rhythm, atrioventricular block, or ventricular block. Finally, for the Seattle Angina Pectoris Questionnaire (SAQ), the standard score = (actual score − lowest score in this area)/(highest score in this area − lowest score in this area). The higher the score, the better the quality of life and functional status of the patient, and the efficacy evaluation using the SF-36 was the same as the SAQ.

### 2.8 Sample size estimation

This study adopted a design of superiority. According to the relevant literature, stable angina with intermingled phlegm and blood stasis syndrome is usually treated for 6 months and the frequency of angina attacks in the conventional Western medicine treatment group was 83 ± 22 times per month. We assumed that compared to the conventional Western medicine treatment group, the traditional Chinese medicine treatment would reduce angina attacks by 7.6 times per month. Assuming that the two sets of standard deviations were the same, we calculated it to be 22, *α* = 0.05, 1 − *β* = 0.9. The sample size estimation formula for comparing the two groups was as follows:

$$n_{T}=\frac{{\left(Z_{1-\alpha }+Z_{1-\beta }\right)}^{2}\delta^{2}(1+1/K)}{{\left(\mu_{T}-\mu_{C}-\Delta \right)}^{2}}$$

The number of cases calculated was 266; thus, with a controlled dropout rate below 12.5%, the number of samples to be observed for both groups should not be less than 304.

### 2.9 Statistical analysis

All data were recorded in the case report forms and then inputted in the form of “double input” and stored in the electronic data collection system (V1.2, Tianjin Zhiyu Future Technology Co., Ltd.). The data were analyzed using R studio version 4.2.1, SAS version 9.4, and SPSS version 26.0. All statistical tests were conducted using a bilateral test, and a *P*-value ≤0.05 was considered statistically significant for the difference being tested. Normally distributed quantitative data were presented as mean ± standard deviation and analyzed by *t*-test and variance analysis; non-normally distributed quantitative data were presented as median and interquartile range and analyzed using the Wilcoxon rank sum test. The confidence intervals for the mean differences are provided. The classification indicators were described using the number of cases and percentages, and the chi-square test was used for inter-group comparison. All statistical tests were conducted using a two-sided test. According to the intention-to-treat (ITT) principle, there were three analysis sets: the full analysis set (FAS), the per-protocol analysis set (PPS), and the safety analysis set (SS). Patients who had been randomized into groups and taken at least one study drug with post-medication evaluation data were included in the FAS, the missing data in the efficacy-related part of the FAS were supplemented using the method of carrying forward the last observation data recorded. All the cases that met the protocol and had complete data were included in the PPS. Patients who had been randomized into groups and taken at least one study drug with post-medication safety evaluation data were included in the SS. The validity result was based on the FAS and PPS. The safety result was based on the SS.

## 3 Results

### 3.1 Patient population

**Figure 1:**
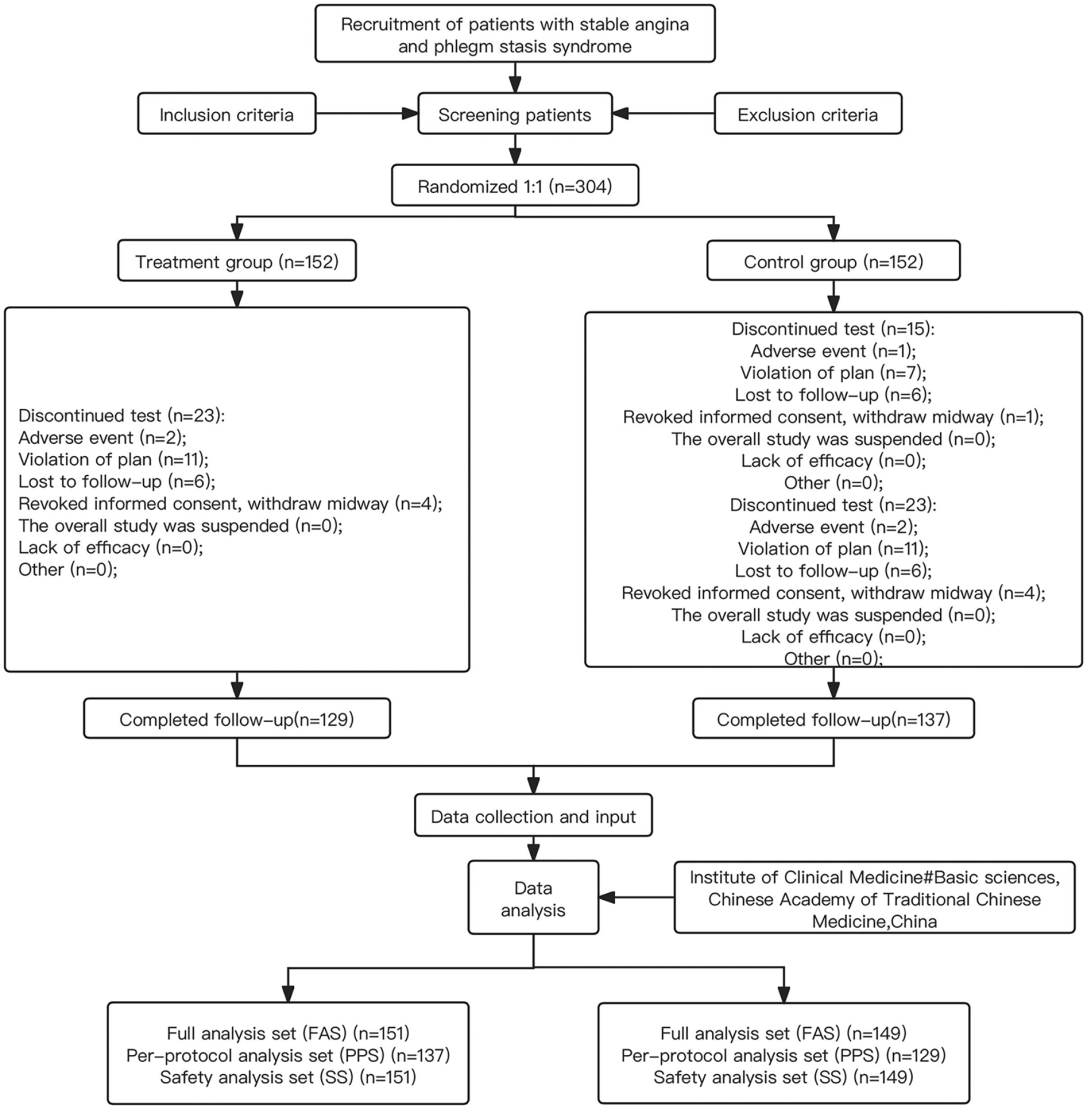
Flow chart of patient enrollment and statistical population division.

Between 10 October 2019 and 13 August 2020, a total of 304 subjects were enrolled, and 300 cases were included in the FAS, 266 cases in the PPS, and 300 cases in the SS (Figure 1, Supplementary Material S1). There were no statistically significant differences between the treatment group and the control group in terms of gender, nationality, age, weight, body mass index (BMI), drinking alcohol, history of drug allergies, family history, cardiovascular diseases, endocrine and metabolic diseases, respiratory system diseases, and treatment history (*P* > 0.05). There were statistically significant differences between the treatment group and the control group for smoking and endocrine and metabolic diseases (*P* < 0.05) (indicated in Table 1). Since the central randomization system was used to generate static block random sequences in this study, the subjects entered the corresponding group according to the assigned random sequence. The centralized random sequence distribution was applied to achieve the principle of distribution concealment. The administrator of the random center did not participate in any part of the trial implementation or follow-up to reduce selectivity bias. Therefore, the imbalance in the number of patients who were smokers and had endocrine and metabolic diseases between the two groups might have been caused by a random error. Thus, the two groups should be considered comparable.

**Table 1:** Main demographic and clinical features of the two groups.

Variable		Treatment group (*n* = 151)	Control group (*n* = 149)	*P*-value
Gender	Male	104 (68.9%)	103 (69.1%)	0.962^a^
	Female	47 (31.1%)	46 (30.9%)	
Nationality	Han	147 (97.4%)	149 (100.0%)	0.123^b^
	Others	4 (2.6%)	0 (0.0%)	
Age (years)	35–44	8 (5.3%)	8 (5.4%)	0.063^a^
	45–59	38 (25.2%)	56 (37.6%)	
	60–75	105 (69.5%)	85 (57.0%)	
Height (cm)		166.51 ± 7.77	165.81 ± 7.74	0.623^c^
Weight (kg)		68.65 ± 11.35	69.31 ± 11.60	0.651^c^
BMI		24.66 ± 3.02	25.12 ± 3.22	0.357^c^
Smoking (%)	Yes	56 (37.1%)	37 (24.8%)	0.022^a^
	No	95 (62.9%)	112 (75.2%)	
Drinking (%)	Yes	40 (26.5%)	39 (26.2%)	0.951^a^
	No	111 (73.5%)	110 (73.8%)	
History of drug allergies (%)	Yes	16 (10.6%)	18 (12.1%)	0.685^a^
	No	135 (89.4%)	131 (87.9%)	
Family history (%)	Yes	39 (25.8%)	36 (24.2%)	0.739^a^
	No	112 (74.2%)	113 (75.8%)	
Cardiovascular diseases (%)	Yes	99 (79.2%)	104 (79.4%)	0.970^a^
	No	26 (20.8%)	27 (20.6%)	
Endocrine and metabolic diseases (%)	Yes	54 (43.2%)	82 (62.6%)	0.002^a^
	No	71 (56.8%)	49 (37.4%)	
Respiratory diseases (%)	Yes	4 (3.2%)	5 (3.8%)	>0.999^b^
	No	121 (96.8%)	126 (96.2%)	
Treatment history (%)	Yes	150 (99.3%)	147 (98.7%)	0.621^b^
	No	1 (0.7%)	2 (1.3%)	

Values are given as number of patients (%) or mean ± SD.

^a^ *P*-value is from Pearson's chi-squared test.

^b^ *P*-value is from Fisher's exact rest for count data.

^c^ *P*-value is from the Wilcoxon rank sum test.

### 3.2 Main efficacy indicators

**Figure 2:**
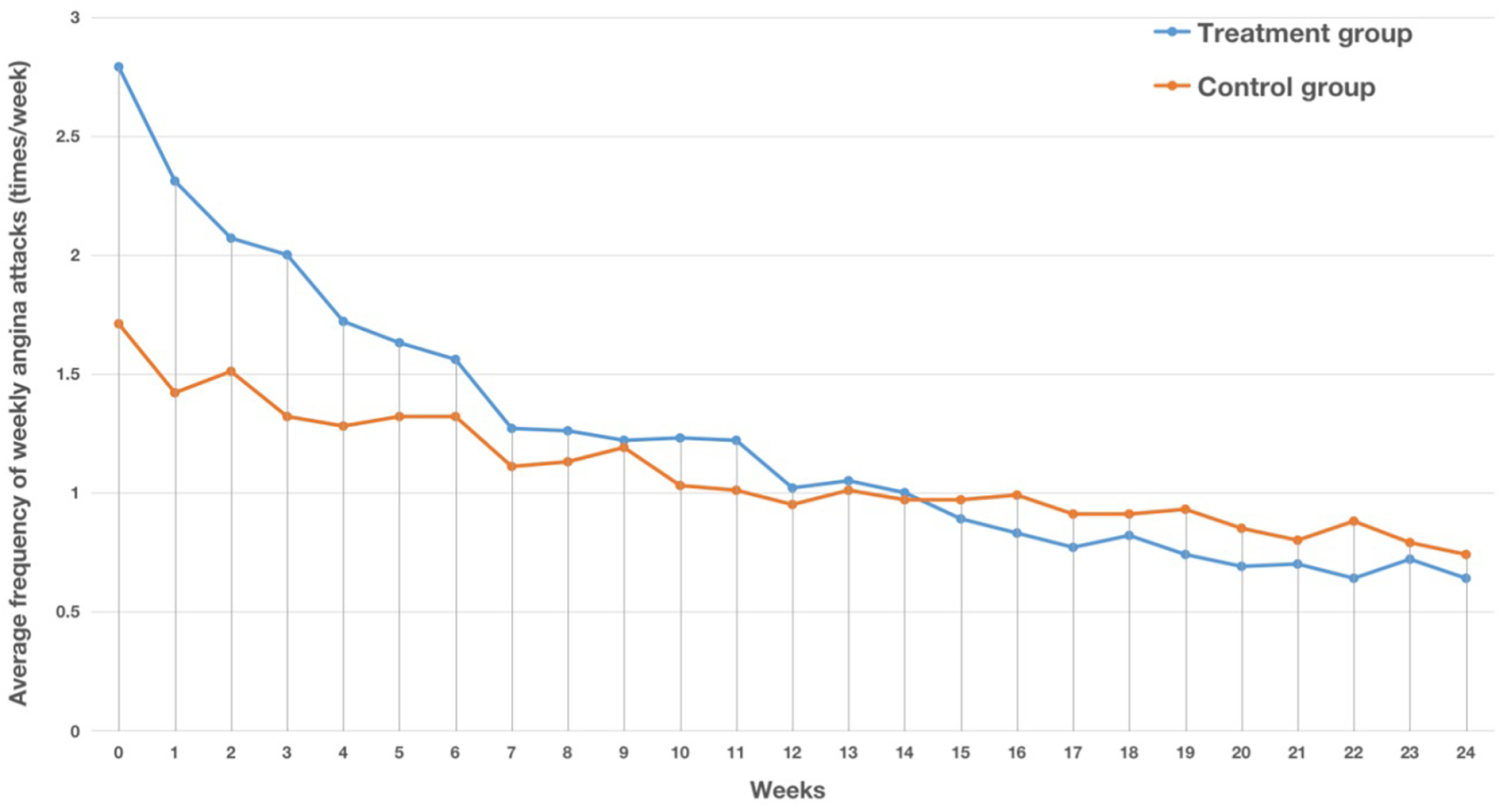
Trend chart of the average number of weekly angina attacks in the two groups in the FAS. The horizontal axis represents the number of weeks, while the vertical axis represents the average number of angina attacks per week.

After treatment, the frequency of weekly angina attacks in both the treatment and control groups was significantly reduced (*P* < 0.05). Before treatment, the frequency of weekly angina attacks in the treatment group was significantly higher than that in the control group, while after treatment, the frequency of weekly angina attacks in the treatment group was significantly lower than that in the control group, and the reduction in the frequency of weekly angina attacks in the treatment group was significantly greater than that in the control group (*P* < 0.05). This indicated that the combination of modified Danlou tablets and conventional Western medicine treatment has advantages over Western medicine treatment alone in reducing the frequency of weekly angina attacks (Table 2, Figure 2). The results of the FAS and PPS were consistent.

**Table 2:** Frequency of weekly angina attacks in the treatment and control groups.

Analysis Set	Group	Before treatment (times/week)	After treatment (times/week)
FAS	Treatment group (*n* = 151)	2.79 ± 3.79	2.93 ± 3.94
	Control group (*n* = 149)	0.64 ± 2.96^a,b,c^	0.60 ± 3.09^a,b,c^
PPS	Treatment group (*n* = 137)	1.71 ± 2.37	1.71 ± 2.48
	Control group (*n* = 129)	0.74 ± 1.22^a^	0.62 ± 1.15^a^

FAS, full analysis set; PPS, per-protocol analysis set.

Kolmogorov–Smirnov test was used, *P* < 0.001, and the data did not follow a normal distribution. The Wilcoxon rank sum test was used for inter-group comparison.

^a^ Comparison with before treatment, *P* < 0.05.

^b^ Comparison with the control group, *P* < 0.05.

^c^ Comparison of *P*-value before and after treatment between the two groups, *P* < 0.05.

### 3.3 Secondary efficacy evaluation indicators

After treatment, there was a significant statistical difference in the angina symptom scores between the treatment group and control group (*P* < 0.05, Table 3). The overall response rate of the treatment group was significantly higher than that in the control group, and the results of the FAS and PPS were consistent. In the FAS, the total score and scores of various dimensions for symptoms of angina pectoris in the treatment group were significantly reduced after treatment (*P* < 0.05), while the nitroglycerin dosage score in the control group did not change significantly (*P* > 0.05). In the PPS, the total score and scores of various dimensions for symptoms of angina pectoris in both groups were significantly reduced after treatment (*P* < 0.05). The duration of angina attacks in the treatment group was significantly lower than the control group (*P* < 0.05) in both the FAS and PPS, and the reduction in total angina symptom score, the degree of angina pectoris pain, duration of angina attacks, and number of attacks in the treatment group was significantly greater than that in the control group (*P* < 0.05, Table 4). In terms of the TCM syndrome score, the overall response rate of the treatment group was significantly higher than that in the control group (*P* < 0.05, Table 3), and the results of the FAS and PPS were consistent.

**Table 3:** Efficacy of angina symptom score, TCM syndrome score, and ECG in the treatment group and control group.

	Analysis set	Group	Significant effect	Effective	Ineffective	Aggravated	Overall response rate	*P*-value
Efficacy of angina symptom score	FAS	Treatment group (*n* = 151)	37 (24.5%)	51 (33.8%)	60 (39.7%)	3 (2.0%)	88 (58.3%)	<0.001^a^
		Control group (*n* = 149)	23 (15.4%)	32 (21.5%)	84 (56.4%)	10 (6.7%)	55 (36.9%)	
	PPS	Treatment group (*n* = 137)	36 (26.3%)	50 (36.5%)	48 (35.0%)	3 (2.2%)	86 (62.8%)	<0.001^a^
		Control group (*n* = 129)	23 (17.8%)	31 (24.0%)	66 (51.2%)	9 (7.0%)	54 (41.8%)	
Efficacy of TCM syndrome score	FAS	Treatment group (*n* = 151)	18 (11.9%)	54 (35.8%)	63 (41.7%)	16 (10.6%)	72 (47.7%)	<0.001^a^
		Control group (*n* = 149)	20 (13.4%)	20 (13.4%)	63 (42.3%)	46 (30.9%)	40 (26.8%)	
	PPS	Treatment group (*n* = 137)	4 (2.9%)	54 (39.4%)	63 (46.0%)	16 (11.7%)	58 (42.3%)	<0.001^a^
		Control group (*n* = 129)	1 (0.8%)	20 (15.5%)	63 (48.8%)	45 (34.9%)	21 (16.3%)	
ECG efficacy evaluation	FAS	18 (16.2%)	30 (27.0%)	62 (55.9%)	1 (0.9%)	48 (43.2%)	18 (16.2%)	<0.001^a^
		7 (6.9%)	18 (17.8%)	64 (63.4%)	12 (11.9%)	25 (24.7%)	7 (6.9%)	
	PPS	18 (16.7%)	30 (27.8%)	59 (54.6%)	1 (0.9%)	48 (44.5%)	18 (16.7%)	<0.001^a^
		6 (6.2%)	18 (18.8%)	60 (62.5%)	12 (12.5%)	24 (25.0%)	6 (6.2%)	

FAS, full analysis set; PPS, per-protocol analysis set.

Values are given as number of patients (%).

^a^ *P*-value is from the Wilcoxon rank sum test.

**Table 4:** Total score and scores of various dimensions of symptoms of angina pectoris in the treatment group and control group.

	Treatment group		Control group	
	Before treatment	After treatment	Before treatment	After treatment
FAS	*n* = 151		*n* = 149	
Total score for angina symptoms	6.45 ± 3.23	3.74 ± 2.73^a,b^	5.77 ± 2.87	4.54 ± 3.05^a^
The degree of angina pectoris pain	2.48 ± 1.15	1.63 ± 1.14^a,b^	2.27 ± 0.98	1.88 ± 1.10^a^
Duration of angina attacks	2.42 ± 1.24	1.54 ± 1.07^a,b,c^	2.26 ± 1.19	1.91 ± 1.30^a^
Number of attacks	1.17 ± 1.25	0.42 ± 0.88^a,b^	0.93 ± 1.20	0.52 ± 0.97^a^
Nitroglycerin dosage	0.38 ± 0.97	0.15 ± 0.52^a^	0.32 ± 0.74	0.23 ± 0.64
PPS	*n* = 137		*n* = 129	
Total score for angina symptoms	6.57 ± 3.25	3.64 ± 2.69^a,b^	5.74 ± 2.87	4.29 ± 2.93^a^
The degree of angina pectoris pain	2.53 ± 1.14	1.61 ± 1.13^a,b^	2.26 ± 0.98	1.80 ± 1.09^a^
Duration of angina attacks	2.42 ± 1.17	1.49 ± 1.00^a,b,c^	2.26 ± 1.16	1.84 ± 1.24^a^
Number of attacks	1.21 ± 1.27	0.39 ± 0.87^a,b^	0.91 ± 1.17	0.48 ± 0.96^a^
Nitroglycerin dosage	0.41 ± 1.00	0.15 ± 0.52^a^	0.29 ± 0.71	0.17 ± 0.56^a^

FAS, full analysis set; PPS, per-protocol analysis set.

Kolmogorov–Smirnov test was used, *P* < 0.001, and the data did not follow a normal distribution. Wilcoxon rank sum test was used for inter-group comparison.

^a^ Comparison with before treatment, *P* < 0.05.

^b^ Comparison of *P*-value before and after treatment between the two groups, *P* < 0.05.

^c^ Comparison with the control group, *P* < 0.05.

**Figure 3:**
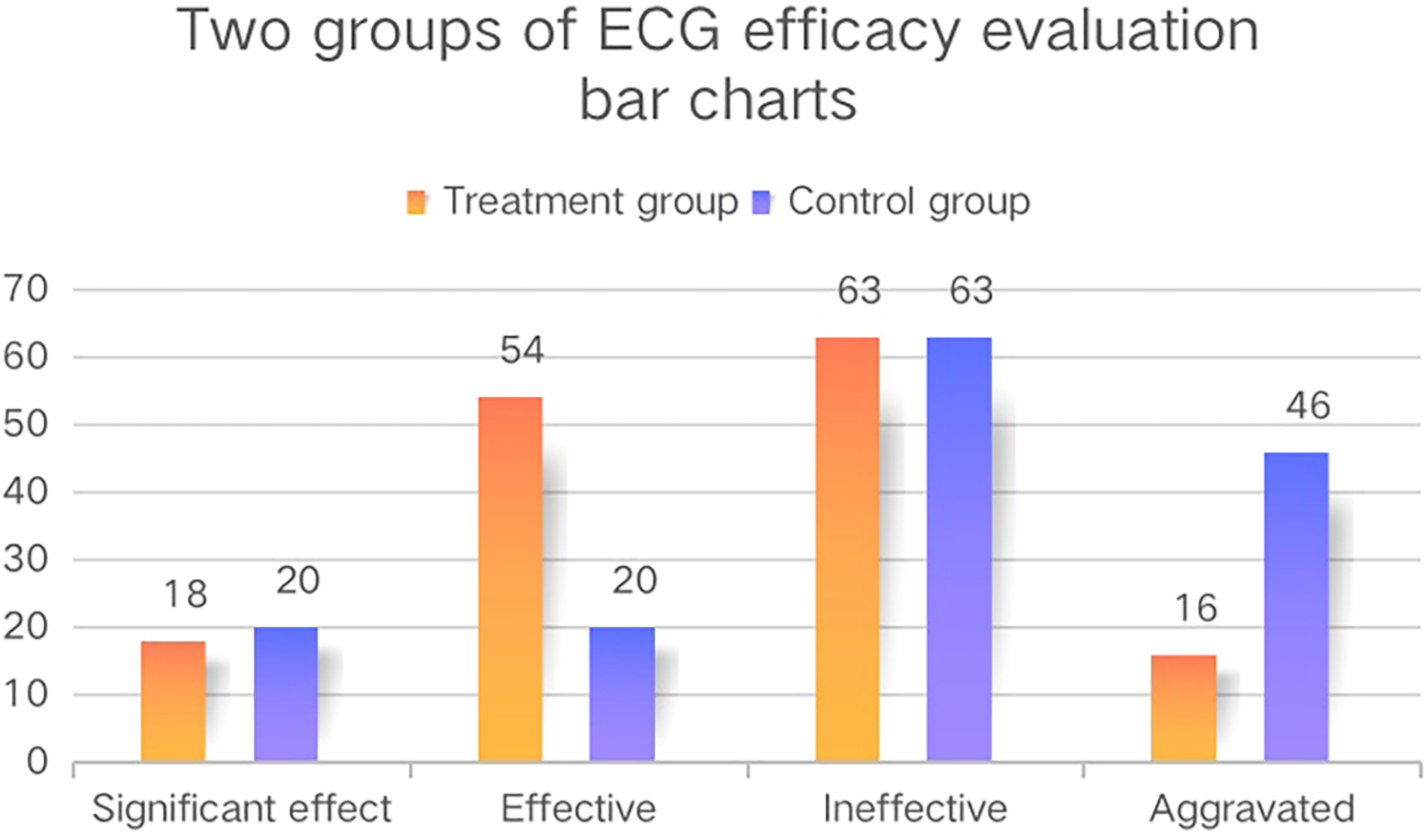
The ECG efficacy evaluation bar charts of the two groups. These bar charts show the ECG efficacy evaluation of the two groups in the FAS and the vertical axis represents the number of patients.

In terms of ECG efficacy evaluation, the overall response rate of the treatment group was significantly higher than that in the control group (*P* < 0.05, Table 3, Figure 3) in both the FAS and PPS. There was no statistically significant difference in the levels of LDL-C, HDL-C, TC, and TG between the two groups of patients in the FAS before and after treatment (*P* > 0.05), while there was a statistically significant difference in the levels of TG between the two groups of patients in the PPS: the level of TG in the treatment group was significantly lower than that in the control group after treatment (*P* < 0.05, Table 5). The level of activated partial thromboplastin time (APTT) in the treatment group in the PPS was significantly reduced after treatment (*P* < 0.05, Table 5), while the changes in blood concretion items [prothrombin time (PT), APTT, TT, and fibrinogen (FIB)] were not significant in the control group before and after treatment. There was no statistically significant difference in the levels of blood concretion items between the two groups of patients in the FAS before and after treatment (*P* > 0.05). In terms of Hcy level, the level of Hcy in the treatment group was significantly reduced after treatment (*P* < 0.05, Table 6), while it did not significantly change in the control group (*P* > 0.05), and the results of the FAS and PPS were consistent. There was no statistically significant difference in the cardiac Doppler two-dimensional ultrasound, blood glucose, hemorheology indicators, or hs-CRP or CRP indicators between the two groups of patients in both the FAS and PPS before and after treatment. Due to the fact that performing an electrocardiogram treadmill exercise test required a doctor's evaluation and the signing of an informed consent form, the number of patients who underwent this test was relatively small and the data from the electrocardiogram treadmill exercise tests were not analyzed.

**Table 5:** The levels of blood lipids and blood concretion items in the two groups in the PPS.

Blood lipids	Treatment group (*n* = 137)		Control group (*n* = 129)	
	Before treatment	After treatment	Before treatment	After treatment
CHOL (mmol/L)	3.85 ± 0.98	3.94 ± 0.95	4.13 ± 1.22	4.21 ± 1.14
TG (mmol/L)	1.65 ± 0.93	1.59 ± 0.93^a^	1.97 ± 3.00	2.02 ± 3.03
LDL-C (mmol/L)	2.16 ± 0.84	2.29 ± 0.82	2.28 ± 1.03	2.28 ± 0.82
HDL-C (mmol/L)	1.18 ± 0.26	1.22 ± 0.31	1.22 ± 0.29	1.25 ± 0.31
PT (s)	11.64 ± 1.37	11.68 ± 1.58	11.75 ± 2.36	11.66 ± 2.27
TT (s)	17.46 ± 1.92	17.16 ± 1.67	17.60 ± 3.20	17.38 ± 1.64
FIB (g/L)	3.10 ± 0.66	3.03 ± 0.68	3.10 ± 0.75	2.93 ± 0.60
APTT (s)	29.52 ± 5.79	30.40 ± 6.50^b^	29.22 ± 6.03	29.32 ± 6.14

CHOL, cholesterol; TG, triglyceride; LDL-C, low-density lipoprotein cholesterol; HDL-C, high-density lipoprotein cholesterol; PT, prothrombin time; TT, thrombin time; FIB, fibrinogen; APTT, activated partial thromboplastin time.

Values are given as mean ± SD. Kolmogorov–Smirnov test was used, *P* < 0.001, and the data did not follow a normal distribution. The Wilcoxon rank sum test was used for the inter-group comparison. Because there is no statistically significant difference in the levels of blood lipids between the two groups of patients in the FAS before and after treatment, the table just shows the levels of blood lipids and blood concretion items of the two groups in the PPS.

^a^ Comparison with the control group, *P* < 0.05.

^b^ Comparison with before treatment, *P* < 0.05.

**Table 6:** The levels of Hcy in the treatment group and control group.

Analysis set	Group	Before treatment (μmol/L)	After treatment (μmol/L)
FAS	Treatment group (*n* = 151)	14.42 ± 7.29	13.70 ± 9.08^a^
	Control group (*n* = 149)	14.83 ± 9.23	14.55 ± 9.86
PPS	Treatment group (*n* = 137)	14.47 ± 7.44	13.67 ± 9.52^a^
	Control group (*n* = 129)	14.83 ± 9.64	15.01 ± 11.16

Hcy, homocysteine.

Values are given as mean ± SD. Hcy: Kolmogorov–Smirnov test was used, *P* < 0.001, and the data did not follow a normal distribution. The Wilcoxon rank sum test was used for inter-group comparison.

^a^ Comparison with before treatment, *P* < 0.05.

In terms of the SAQ and SF-36, after treatment, the scores in the two groups significantly increased (*P* < 0.05, Table 7). The SAQ and SF-36 scores in the treatment group were significantly higher than those in the control group (*P* < 0.05), and the increase in the scores was significantly greater than that of the control group. The results of the FAS and PPS were consistent.

**Table 7:** The SAQ and SF-36 scores in the treatment group and control group.

	Treatment group		Control group	
	Before treatment	After treatment	Before treatment	After treatment
FAS	*n* = 151		*n* = 149	
SAQ score	76.09 ± 9.96	80.99 ± 8.37^a,b,c^	76.64 ± 8.98	78.30 ± 8.84^a^
SF-36 score	110.12 ± 11.61	124.70 ± 12.29^a,b,c^	111.11 ± 10.08	119.95 ± 13.34^a^
PPS	*n* = 137		*n* = 129	
SAQ score	76.17 ± 9.97	81.47 ± 8.09^a,b,c^	77.09 ± 8.90	78.89 ± 8.70^a^
SF-36 score	109.84 ± 11.67	125.51 ± 11.98^a,b,c^	111.84 ± 10.01	121.61 ± 13.04^a^

FAS, full analysis set; PPS, per-protocol analysis set; SAQ, Seattle Angina Pectoris Questionnaire; SF-36, 36-item Health Status Survey Summary Form.

Kolmogorov–Smirnov test was used, *P* > 0.05, and the data in the two groups followed a normal distribution. A *t*-test was used for the inter-group comparison.

^a^ Comparison with before treatment, *P* < 0.05.

^b^ Comparison with control group, *P* < 0.05.

^c^ Comparison of *P*-value before and after treatment between the two groups, *P* < 0.05.

### 3.4 Safety indicators

In the SS analysis set, there was no statistically significant difference in blood routine, liver function, kidney function, urine routine, and stool routine between the two groups before treatment (*P* > 0.05), and the changes were not significant after treatment (*P* > 0.05) (Additional Material S2).

Among the 300 patients in the SS analysis set, 36 experienced adverse events, including 15 cases (10.3%) in the control group and 21 cases (13.9%) in the treatment group. There was no statistically significant difference between the two groups (*P* > 0.05). Among the 17 patients with adverse events in the control group, 3 (17.6%) experienced serious adverse events, while in the treatment group, 5 (20%) experienced serious adverse events. There was no statistically significant difference between the two groups (*P* > 0.05).

### 3.5 Long-term follow-up

In the SS analysis set, after treatment, patients were followed up by telephone in weeks 36 and 48. The results showed that there were no statistically significant differences between the two groups in terms of whether they had comorbidities, the type of comorbidities (differences in endocrine and metabolic system diseases were statistically significant, which was consistent with the difference before treatment), and whether the comorbidities persisted during the follow-up period (*P* > 0.05). There were no cases of revascularization, death, or readmission during the follow-up period.

## 4 Discussion

Our study indicated that the use of Danlou tablets and its modified combination therapy based on conventional Western medicine treatment could significantly reduce the weekly frequency of angina attacks in subjects with stable angina pectoris with IPBS syndrome and its concurrent symptoms; improve the efficacy of angina symptoms, TCM syndrome, and ECG; extend the activated partial thromboplastin time; reduce triglyceride levels and homocysteine levels; and improve Seattle Angina Pectoris Questionnaire and SF-36 scores, and demonstrates advantages over conventional Western medicine treatment, as well as being safe and having a good long-term prognosis. A study conducted a clinical epidemiological survey on 8,129 patients with coronary heart disease from 40 tertiary traditional Chinese medicine or integrated traditional Chinese and Western medicine hospitals in 21 provinces, cities, and autonomous regions of China. It found that coronary heart disease was usually characterized by a deficiency in origin and excess in superficiality: the deficiency in origin mainly resulted from qi deficiency (67.17%), and the excess in superficiality mainly resulted from blood stasis (77.89%) and phlegm turbidity (43.97%). With the changes in the disease spectrum and patient lifestyle, the traditional Chinese medicine syndromes of coronary heart disease are becoming more complex, showing a trend of multiple syndrome elements that coexist to cause disease. Single or composite syndromes based on phlegm turbidity and blood stasis might continue to rise in the future, and phlegm and blood stasis might become the main syndrome type for coronary heart disease (14, 15). Danlou tablets, the only listed traditional Chinese patent medicine recommended in the Chinese Medicine Diagnosis and Treatment Guide for Stable Angina Pectoris of Coronary Heart Disease for the treatment of intermingled phlegm and blood stasis syndrome in stable angina pectoris, were effective in reducing the incidence of major cardiovascular events, improving mobility, reducing the number of angina attacks and TCM syndromes, improving coronary microcirculation disorders, reducing the incidence of angina pectoris after percutaneous coronary intervention (PCI), and reducing the use of nitroglycerin (16–19).

However, the syndrome of intermingled phlegm and blood stasis in stable angina does not exist in isolation and is often accompanied by concurrent symptoms such as qi deficiency, qi stagnation, and toxin accumulation. Therefore, our study focused on the syndrome of intermingled phlegm and blood stasis in stable angina pectoris by setting strict inclusion and exclusion standards, accurately locating the suitable population, and dividing the research population into four parts based on TCM characteristics based on the dialectical thinking of the “Differentiation of symptoms and signs based on symptoms and combination of diseases and syndromes”: IPBS syndrome, IPBS-QD syndrome, IPBS-QS syndrome, and IPBS-TA syndrome. In terms of intervention measures, the control group received routine Western medicine treatment, while the experimental group received Danlou tablets in addition to the treatment received by the control group. In addition to the intervention plan for the main syndrome of intermingled phlegm and blood stasis, symptomatic intervention measures were adopted for the concurrent syndromes. For those who experienced qi deficiency, *C. pilosula*, *P. cocos*, and *P. ternata* were added to enhance the ability of Danlou tablets to tone qi, strengthen the spleen, activate the yang, and discharge turbidity. For those who experienced qi stagnation, *R. corydalis*, *F. aurantii immaturus*, and *P. ternata* were added to enhance the ability of Danlou tablets to regulate qi, calm the pain, and dissipate the phlegm and nodules. For those who experienced toxin accumulation, *R. officinale*, *P. cuspidatum*, and *P. ternata* were added to enhance the ability of Danlou tablets to promote blood circulation, detoxify, and dissipate phlegm and nodules. These interventions took into account both the standardization of group therapy and the flexibility of individual therapy, fully embodying the characteristic TCM clinical thinking of “Fully understanding the essence of diseases and corresponding prescriptions and syndromes.” Our study adopted a randomized controlled design, and the intervention measures were relatively flexible while ensuring repeatability. This was more in line with the characteristics of dynamic syndrome differentiation and treatment in TCM and met the practical needs of clinical diagnosis and treatment in TCM. In terms of data analysis, the statistical result analysis adopted the ITT principle, paid attention to missing data, combined the FAS and PPS results, and the PPS results were used to test the robustness of the test results to objectively evaluate their clinical significance and ensure the test results better reflected the actual clinical effect (20).

The research results of Park et al. suggested that Danlou tablets can improve the synthesis and release of NO, thereby increasing the oxygen supply and tissue perfusion of endothelial cells. This may be one of the important mechanisms of Danlou tablets in treating coronary heart disease. Therefore, the therapeutic advantage of Danlou tablets in reducing the frequency of weekly angina attacks and improving the effective rate of ECG and angina symptom scores might be related to the effect of Danlou tablets in increasing No levels, thereby expanding blood vessels, and improving myocardial blood supply (21). It was reported that high levels of Hcy can increase the clearance rate of high-density lipoprotein by inhibiting the synthesis of apoA1 (22), thereby reducing the reverse transport of HDL-C to serum cholesterol. Furthermore Hcy self-oxidation can cause oxidative modification of low-density lipoprotein, thus increasing the uptake of oxidized low-density lipoprotein by mega phage cells, causing lipid deposition in the vascular wall and coronary atherosclerosis (23). In addition, high concentrations of Hcy could reduce the bioavailability of vascular endothelial protective factor NO, inhibit the expression of oxygenase 1, and lead to endothelial cell damage, leading to endothelial cell proliferation. Moreover, Hcy could activate various inflammatory cells and promote the release of inflammatory mediators, leading to endothelial damage and the occurrence and development of coronary heart disease (24, 25). Therefore, it can be concluded that Danlou tablets might delay the process of coronary atherosclerosis and play a therapeutic role in CHD by reducing the level of homocysteine, thereby improving lipid metabolism, reducing the deposition of cholesterol and triglycerides in the vascular wall, protecting vascular endothelial function, and reducing inflammatory reaction. APTT mainly reflects changes in coagulation factors V, VIII, IX, X, and XI, and is the most sensitive and commonly used screening test indicator for the endogenous coagulation system (26). The detection of activated partial thromboplastin time could predict the pre-thrombotic state and reduce the occurrence of acute coronary syndrome (27). The results of our study indicated that Danlou tablets could significantly prolong the activated partial thromboplastin time, thereby improving the coagulation function status of patients with stable angina pectoris with intermingled phlegm and blood stasis syndrome. It might have a certain improvement effect by preventing thrombosis and reducing the occurrence of acute cardiovascular events. An elevated serum triglyceride level is an independent risk factor for coronary heart disease. Research has confirmed that small dense low-density lipoprotein (sLDL) is the main carrier of triglycerides, and when triglycerides increase, under the action of cholesterol TP, cholesterol in low-density lipoprotein is transferred to extremely low-density lipoprotein, and triglycerides in sLDL are transferred to low-density lipoprotein although the total amount of LDL remains unchanged. When the triglycerides in low-density lipoprotein protein increase to a certain extent, some are hydrolyzed by liver lipase, causing the particles of low-density lipoprotein to become sLDL (28). sLDL produced by hypertriglyceridemia is an important factor in atherosclerosis and promotes the rupture of atherosclerotic plaque. It is easily ingested by macrophages to produce an oxidation reaction and local inflammatory reaction, which cause excessive cholesterol esters to gather in cells and generate foam cells, which become the main component of atherosclerotic plaque and promotes the formation or rupture of atherosclerotic plaque (29, 30). Therefore, it could be inferred that Danlou tablets might have the effect of delaying coronary atherosclerosis and stabilizing coronary atherosclerotic plaque, which is achieved by reducing the serum triglyceride level and thus the sLDL level. In addition, the therapeutic advantages of Danlou tablets demonstrated by the SAQ and SF-36 scores also indicated that Danlou tablets can improve the quality of life and physical and mental health of stable angina patients with IPBS and its concurrent symptoms from two aspects: physiological health and psychological health.

A clinical trial by Wang et al. showed that treatment with Danlou tablets could alleviate inflammatory activation by reducing the serum level of hs-CRP, soluble CD40, and interleukin-6 (31). However, our study demonstrated that there was no significant difference in the serum level of hs-CRP between the two groups. The patients included in this study were all stable angina pectoris patients, who were in a stable stage of disease after ischemic cardiomyopathy and acute coronary syndrome (≥6 months after onset). Among the 304 subjects included in this study, 253 (83.2%) had normal hs-CRP/CRP levels, 25 had abnormal and clinically insignificant levels, and only 8 had abnormal and clinically significant levels. The presence of a large number of patients within the normal level range might have led to statistically insignificant differences between the before and after treatment levels. However, hs-CRP and CRP have high sensitivity and low specificity and are susceptible to many factors. Among the eight subjects with abnormal clinical significant levels, the clinical significance description was mostly cold and cough, lung infection, and urinary system infection, which also had a certain impact on the test conclusion.

There were some limitations in this study. Because of the particularity of randomized controlled trials for the practicality of traditional Chinese medicine, the participants and physicians could not be blinded; thus, the study was not designed as a double-blind placebo-controlled trial, and a placebo was not routinely used in the effectiveness evaluation (32, 33). At the same time, the use of a placebo has limited benefits for evaluating independent measurements of continuous experimental results (34). Therefore, we blinded the statistical analysts and medical technology testing personnel to objectively evaluate efficacy indicators and test results, to minimize detection errors and subjective efficacy evaluation bias. Due to limitations in funding and research time, no dynamic intervention based on TCM was conducted. Our next step will be to understand the advantages of the intervention measures in terms of efficacy and select the ideal time point for the target population to carry out efficacy observations of dynamic interventions based on TCM indications. In addition, this report only conducted a comparative analysis of the therapeutic effects between the experimental group and the control group and did not consider the effects of different subgroups (IPBS group, IPBS-QD group, IPBS-QS group, and IPBS-TA group), age, gender, BMI, subcenter, and concurrent diseases on the outcome indicators. At a later stage, we will conduct subgroup analysis on the above factors to comprehensively evaluate the clinical efficacy of Danlou tablets and their modified treatments.

## 5 Conclusion

The use of Danlou tablets and the modified combination therapy based on conventional Western medicine treatment could significantly reduce the weekly frequency of angina attacks in patients with stable angina pectoris with IPBS syndrome and its concurrent symptoms; improve the efficacy of angina symptoms, TCM syndromes, and ECG; extend the activated partial thromboplastin time; reduce triglyceride and homocysteine levels; and improve the patient’s SAQ and the SF-36 scores. Furthermore, the Danlou tablet treatment and the combination therapy had good clinical efficacy, safety, and long-term prognosis, and are worth further promotion and application in clinical practice. Further basic experiments should be designed to verify its mechanism of action.

## Data Availability

The raw data supporting the conclusions of this article will be made available by the authors, without undue reservation.
