# Supplementary material for: Efficacy and safety of Danlou tablets in the treatment of stable angina pectoris with intermingled phlegm and blood stasis syndrome in coronary heart disease: a multicenter randomized controlled study
Source: Front Cardiovasc Med. 2024 Oct 30;11:1462730. doi: 10.3389/fcvm.2024.1462730 (PMC11571080; doi:10.3389/fcvm.2024.1462730)
Supplement: Supplementary file 1 [file Datasheet1.pdf]

Intermingled Phlegm and Blood Stasis (IPBS): Stable angina belongs to the syndrome of intrinsic deficiency and standard deficiency, with qi deficiency as the main symptom, and qi stagnation, phlegm turbidity, blood stasis, fire (heat) as the standard. Qi deficiency, phlegm turbidity, and blood stasis syndrome elements run through the entire pathological process of stable angina. This disease is more common in middle-aged and elderly people. After reaching middle and old age, the function of the internal organs weakens, the qi is depleted, and in the early stage of the disease, qi deficiency leads to insufficient blood circulation and fluid circulation. Blood stagnation and fluid stagnation stop and turn into phlegm and blood stasis, obstructing the heart and blood vessels; In addition, with the acceleration of modern life pace and the increase of social, work, and mental pressure, it is easy to cause internal depression of the seven emotions, stagnation of qi mechanism, loss of liver function, and obstruction of the heart meridian, resulting in chest obstruction. Therefore, in the early stage of the attack, it is common to have qi deficiency, qi stagnation, and chest obstruction. By the middle stage of the disease, phlegm turbidity and blood stasis have formed. Phlegm turbidity and blood stasis are not only pathological products of qi deficiency and qi stagnation, but also pathogenic factors of stable angina pectoris. They can cause a single disease or be combined with other diseases. The "Blood Syndrome Theory" states: "Phlegm can also turn into blood stasis" and "blood accumulation can also turn into phlegm water". The physiological homology between body fluids and blood constitutes the inevitability of "phlegm blood stasis correlation", that is, the Intermingled Phlegm and Blood Stasis (IPBS).

Statistical analysis of population segmentation

Randomized population: All randomized subjects constitute the randomized population. According to the intention to treat (ITT) principle, statistical analysis will be conducted on all randomized subjects.

(1) Full Analysis Set (FAS):

All randomized cases that have taken the study drug at least once and have post medication evaluation data constitute the full analysis set of this study. The missing data in the efficacy related section of the full analysis will be supplemented using the method of carrying forward the last observation data. The full analysis set is the main population for evaluating the efficacy of this study.

(2) Per Protocol (PP) population:

The criteria for the PP population set and its population will be ultimately determined during data verification, including at least the following criteria:

a. Meet the selection criteria specified in the experimental protocol;

b. The main observation indicators have baseline data;

c. Complete all planned visits;

d. During the trial period, no drugs or treatments that may affect the efficacy evaluation were used;

e. Good compliance (80% -120%).

(3) Safety population:

All randomized cases that have taken the study drug at least once and have safety evaluation data after medication constitute the safety population of this study. The safety population is the main group for safety evaluation in this study.
