# Supplementary material for: Efficacy and safety of Danlou tablets in the treatment of stable angina pectoris with intermingled phlegm and blood stasis syndrome in coronary heart disease: a multicenter randomized controlled study
Source: Front Cardiovasc Med. 2024 Oct 30;11:1462730. doi: 10.3389/fcvm.2024.1462730 (PMC11571080; doi:10.3389/fcvm.2024.1462730)
Supplement: Supplementary file 2 [file Datasheet2.pdf]

The incidence of adverse events

The number, numbers and incidence of all adverse events, related adverse events, serious adverse events, severe adverse events, and severe adverse events related to the study drug were calculated respectively, and analyzed by χ 2 test or Fisher's exact test.

Adverse events will be classified by System Organ Class (SOC, System Organ Classification) and Preferred Term (PT, Preferred Term). List of adverse events related to study drug, list of adverse events unrelated to study drug, list of adverse events leading to shedding, and list of serious adverse events, respectively.

1.1 Absence of adverse events

variable	Test group (N=151)	Control group (N=149)	Total (N=300)	method of calibration	statistics	P price
Any adverse events				Pearson's Chi-squared test	X-squared = 0.92	0.337
have	21 (13.9%)	15 (10.3%)	36 (12.1%)			
not have	130 (86.1%)	131 (89.7%)	261 (87.9%)			
Missing	0	3	3			

1.2 Specific description of the adverse events

Subjects number	Adverse event description	Whether to continue	order of severity	Association with the study drug	Measures taken for the study drug	lapse to	whether or not SAE	group
010016	Labor angina pectoris	deny	severe	Certainly irrelevant	There was no change in dose	Recovery, without sequelae	yes	test team

Subjects number	Adverse event description	Whether to continue	order of severity	Association with the study drug	Measures taken for the study drug	lapse to	whether or not SAE	group
010017	unstable angina	deny	severe	It may not matter	Permanent withdrawal of drugs	Recovery, without sequelae	yes	test team
020026	Elevation of the homocysteine profile	yes	mild	Certainly irrelevant	not applicable	Ongoing, with continued follow-up	deny	control group
030009	Urinary leukocytes were positive	yes	mild	Certainly irrelevant	There was no change in dose	Ongoing, with continued follow-up	deny	test team
050006	Inthrombus thrombosis in the LV wall	deny	mild	Certainly irrelevant	not applicable	Recovery, without sequelae	yes	control group
050012	diarrhoea	deny	mild	Probably about	There was no change in dose	Recovery, without sequelae	deny	test team
050024	Positive urine sugar	deny	mild	Certainly irrelevant	not applicable	Recovery, without sequelae	deny	control group
070010	dyslipidemia	yes	mild	It may not matter	There was no change in dose	Ongoing, with continued follow-up	deny	test team
070011	Prostate malignancy	yes	severe	Certainly irrelevant	not applicable	Ongoing, with continued follow-up	yes	control group
070020	Urinary latent blood was positive	deny	mild	Certainly irrelevant	not applicable	Recovery, without sequelae	deny	control group

Subjects number	Adverse event description	Whether to continue	order of severity	Association with the study drug	Measures taken for the study drug	lapse to	whether or not SAE	group
080003	Subactive blood positive	deny	severe	It may not matter	Suspension of medication	Recovery, without sequelae	yes	test team
080009	Higher fasting blood glucose and positive urinary glucose	yes	mild	Certainly irrelevant	not applicable	Recovery, without sequelae	deny	control group
080020	Headache caused by isosorbide mononitrate tablets	deny	mild	Certainly irrelevant	not applicable	Recovery, without sequelae	deny	test team
090005	stomach ache	deny	mild	It's likely to be relevant	Permanent withdrawal of drugs	Recovery, without sequelae	deny	test team
100003	Hypertriglyceridemia	yes	mild	It may not matter	There was no change in dose	Ongoing, with continued follow-up	deny	test team
100005	hypertriglyceridemia	deny	mild	Certainly irrelevant	There was no change in dose	Recovery, without sequelae	deny	test team
100008	Cholerol and triglycerides increased	yes	mild	Certainly irrelevant	not applicable	Ongoing, with continued follow-up	deny	control group

Subjects number	Adverse event description	Whether to continue	order of severity	Association with the study drug	Measures taken for the study drug	lapse to	whether or not SAE	group
100011	hypercholesterolemia	yes	mild	Certainly irrelevant	There was no change in dose	Ongoing, with continued follow-up	deny	test team
100012	hypertriglyceridemia	yes	mild	Certainly irrelevant	not applicable	Ongoing, with continued follow-up	deny	control group
100014	hypertriglyceridemia	yes	mild	It may not matter	There was no change in dose	Ongoing, with continued follow-up	deny	test team
100015	hypertriglyceridemia	deny	mild	Certainly irrelevant	There was no change in dose	Recovery, without sequelae	deny	test team
100020	Elevation of the homocysteine profile	yes	mild	It may not matter	There was no change in dose	Ongoing, with continued follow-up	deny	test team
100021	hematencephalon	deny	mild	Certainly irrelevant	not applicable	Recovery, without sequelae	yes	control group
110008	C and F	deny	mild	It may not matter	There was no change in dose	Recovery, without sequelae	deny	test team
110009	Hemoglobin values were too low	yes	mild	Certainly irrelevant	There was no change in dose	Ongoing, with continued follow-up	deny	test team
110012	urinary tract infection	yes	mild	Certainly irrelevant	not applicable	Ongoing, with continued follow-up	deny	control group

Subjects number	Adverse event description	Whether to continue	order of severity	Association with the study drug	Measures taken for the study drug	lapse to	whether or not SAE	group
110013	Medium and low cut viscosity in whole blood	yes	mild	It may not matter	There was no change in dose	Recovery, without sequelae	deny	test team
110014	The cut viscosity was increased in whole blood	deny	mild	Certainly irrelevant	not applicable	Recovery, without sequelae	deny	control group
110015	Indirect bilirubin was elevated	yes	mild	Certainly irrelevant	not applicable	Ongoing, with continued follow-up	deny	control group
110017	C and F	deny	mild	Certainly irrelevant	not applicable	Recovery, without sequelae	deny	control group
110018	hyperlipemia	yes	mild	Certainly irrelevant	There was no change in dose	Ongoing, with continued follow-up	deny	test team
110019	C and F	deny	mild	Certainly irrelevant	not applicable	Recovery, without sequelae	deny	control group
110021	Higher hypocut viscosity in whole blood	yes	mild	It may not matter	There was no change in dose	Recovery, without sequelae	deny	test team
110022	angina pectoris	deny	severe	It may not matter	There was no change in dose	Ongoing, with continued follow-up	yes	test team
110023	Elevated urinary protein	yes	mild	Certainly irrelevant	not applicable	Recovery, without sequelae	deny	control group

Subjects number	Adverse event description	Whether to continue	order of severity	Association with the study drug	Measures taken for the study drug	lapse to	whether or not SAE	group
120005	Positive fecal occult blood	yes	mild	It may not matter	Suspension of medication	Recovery, without sequelae	deny	test team
100003	Elevated fasting blood glucose	deny	mild	It may not matter	There was no change in dose	Recovery, without sequelae	deny	test team
100014	Platelet values were reduced	deny	mild	It may not matter	There was no change in dose	Ongoing, with continued follow-up	deny	test team
100015	hypertriglyceridemia	yes	mild	Certainly irrelevant	There was no change in dose	Ongoing, with continued follow-up	deny	test team
110015	Elevated cholesterol	yes	mild	Certainly irrelevant	not applicable	Ongoing, with continued follow-up	deny	control group
110017	TT decreased and FIB increased	yes	mild	Certainly irrelevant	not applicable	Ongoing, with continued follow-up	deny	control group
100014	unstable angina	deny	moderate	It may not matter	There was no change in dose	Recovery, without sequelae	yes	test team

1.3 Severity

variable	Test group (N=25)	Control group (N=17)	Total (N=42)	method of calibration	95%CI	statistics	P price

variable	Test group (N=25)	Control group (N=17)	Total (N=42)	method of calibration	95%CI	statistics	P price
order of severity				Wilcoxon rank sum test	(0 , 0)	T = 183.00	0.221
mild	20 (80.0%)	16 (94.1%)	36 (85.7%)				
moderate	1 (4.0%)	0 (0.0%)	1 (2.4%)				
severe	4 (16.0%)	1 (5.9%)	5 (11.9%)				
Average rank	22.68	19.765	21.5				
Missing	0	0	0				

1.4 whether the SAE

variable	Test group (N=25)	Control group (N=17)	Total (N=42)	method of calibration	P price
whether or not SAE				Fisher's Exact Test for Count Data	> 0.999
yes	5 (20.0%)	3 (17.6%)	8 (19.0%)		
deny	20 (80.0%)	14 (82.4%)	34 (81.0%)		
Missing	0	0	0		

1.5 Description of serious adverse events

Subjects number	Adverse event description	Whether the adverse events persist	order of severity	Association with the study drug	Measures taken for the study drug	lapse to	group

Subjects number	Adverse event description	Whether the adverse events persist	order of severity	Association with the study drug	Measures taken for the study drug	lapse to	group
010016	Labor angina pectoris	deny	severe	Certainly irrelevant	There was no change in dose	Recovery, without sequelae	test team
010017	unstable angina	deny	severe	It may not matter	Permanent withdrawal of drugs	Recovery, without sequelae	test team
050006	Inthrombus thrombosis in the LV wall	deny	mild	Certainly irrelevant	not applicable	Recovery, without sequelae	control group
070011	Prostate malignancy	yes	severe	Certainly irrelevant	not applicable	Ongoing, with continued follow-up	control group
080003	Subactive blood positive	deny	severe	It may not matter	Suspension of medication	Recovery, without sequelae	test team
100021	hematencephalon	deny	mild	Certainly irrelevant	not applicable	Recovery, without sequelae	control group
110022	angina pectoris	deny	severe	It may not matter	There was no change in dose	Ongoing, with continued follow-up	test team
100014	unstable angina	deny	moderate	It may not matter	There was no change in dose	Recovery, without sequelae	test team

Safety indicators

1 Blood routine

1.1 RBC count for RBC

(1) Comparison between the groups of the visiting viewpoints

variable	Test group (N=151)	Control group (N=149)	Total (N=300)	method of calibration	95%CI	statistics	P price
Visit 1_ Red blood cell count RBC				t-test	(-0.148 , 0.055)	t = -0.91	0.366
Mean (SD)	4.72 (0.46)	4.77 (0.42)	4.75 (0.44)				
Median (Q1, Q3)	4.74 (4.36, 5.08)	4.76 (4.49, 5.08)	4.74 (4.43, 5.09)				
Min - Max	3.80 - 6.09	3.70 - 5.95	3.70 - 6.09				
Missing	1	2	3				
P-values for the test of normality	0.187	0.689					
Visit 2_ Red blood cell count RBC				t-test	(-0.165 , 0.053)	t = -1.01	0.315
Mean (SD)	4.74 (0.49)	4.79 (0.38)	4.76 (0.44)				
Median (Q1, Q3)	4.73 (4.39, 5.11)	4.79 (4.53, 5.11)	4.78 (4.44, 5.11)				
Min - Max	3.64 - 5.82	3.76 - 5.56	3.64 - 5.82				
Missing	14	29	43				
P-values for the test of normality	0.074	0.225					

variable	Test group (N=151)	Control group (N=149)	Total (N=300)	method of calibration	95%CI	statistics	P price
Visit 3_ RBC cell count RBC				t-test	(-0.124 , 0.117)	t = -0.06	0.952
Mean (SD)	4.80 (0.52)	4.80 (0.39)	4.80 (0.46)				
Median (Q1, Q3)	4.78 (4.41, 5.17)	4.83 (4.50, 5.11)	4.80 (4.44, 5.13)				
Min - Max	3.68 - 6.06	3.84 - 5.96	3.68 - 6.06				
Missing	27	40	67				
P-values for the test of normality	0.508	0.713					
Visit 4_ Red blood cell count RBC				t-test	(-0.19 , 0.05)	t = -1.14	0.254
Mean (SD)	4.78 (0.53)	4.85 (0.44)	4.81 (0.49)				
Median (Q1, Q3)	4.80 (4.41, 5.11)	4.84 (4.57, 5.11)	4.82 (4.49, 5.11)				
Min - Max	3.36 - 6.30	3.63 - 6.03	3.36 - 6.30				
Missing	20	23	43				
P-values for the test of normality	0.913	0.97					

(2) Baseline and each view group

variable	group	base line	final result	method of calibration	95%CI	statistics	P price
Visit 2_ Red blood cell count RBC	Control group (N=149)			Wilcoxon signed rank test	(-0.05 , 0.04)	T = 3468.5	0.911
	Mean (SD)	4.77 (0.42)	4.79 (0.38)				
	Median (Q1, Q3)	4.76 (4.49, 5.08)	4.79 (4.53, 5.11)				
	Min - Max	3.70 - 5.95	3.76 - 5.56				
	Missing	2	29				
	Test group (N=151)			Wilcoxon signed rank test	(-0.04 , 0.05)	T = 4429.5	0.808
	Mean (SD)	4.72 (0.46)	4.74 (0.49)				
	Median (Q1, Q3)	4.74 (4.36, 5.08)	4.73 (4.39, 5.11)				
	Min - Max	3.80 - 6.09	3.64 - 5.82				
	Missing	1	14				
Visit 3_ RBC cell count RBC	Control group (N=149)			Wilcoxon signed rank test	(-0.09 , 0)	T = 2148.5	0.06
	Mean (SD)	4.77 (0.42)	4.80 (0.39)				
	Median (Q1, Q3)	4.76 (4.49, 5.08)	4.83 (4.50, 5.11)				

variable	group	base line	final result	method of calibration	95%CI	statistics	P price
	Min - Max	3.70 - 5.95	3.84 - 5.96				
	Missing	2	40				
	Test group (N=151)			Wilcoxon signed rank test	(-0.11 , 0.01)	T = 3038.5	0.092
	Mean (SD)	4.72 (0.46)	4.80 (0.52)				
	Median (Q1, Q3)	4.74 (4.36, 5.08)	4.78 (4.41, 5.17)				
	Min - Max	3.80 - 6.09	3.68 - 6.06				
	Missing	1	27				
Visit 4_ Red blood cell count RBC	Control group (N=149)			Paired t-test	(-0.105 , 0.01)	t = -1.632	0.105
	Mean (SD)	4.77 (0.42)	4.85 (0.44)				
	Median (Q1, Q3)	4.76 (4.49, 5.08)	4.84 (4.57, 5.11)				
	Min - Max	3.70 - 5.95	3.63 - 6.03				
	Missing	2	23				
	Test group (N=151)			Wilcoxon signed rank test	(-0.08 , 0.035)	T = 3846	0.416
	Mean (SD)	4.72 (0.46)	4.78 (0.53)				

variable	group	base line	final result	method of calibrati on	95%CI	statistic s	P price
	Median (Q1, Q3)	4.74 (4.36, 5.08)	4.80 (4.41, 5.11)				
	Min - Max	3.80 - 6.09	3.36 - 6.30				
	Missing	1	20				

1.2 Hemoglobin Hb

(1) Comparison between the groups of the visiting viewpoints

variable	Test group (N=151)	Control group (N=149)	Total (N=300)	method of calibration	95%CI	statistics	P price
Visit 1_ Hemoglobin Hb				t-test	(-3.931 , 2.316)	t = -0.51	0.611
Mean (SD)	144.57 (13.45)	145.37 (13.90)	144.97 (13.66)				
Median (Q1, Q3)	145.00 (135.00, 154.00)	145.00 (136.50, 155.00)	145.00 (135.00, 155.00)				
Min - Max	104.00 - 176.00	100.00 - 183.00	100.00 - 183.00				
Missing	1	2	3				
P-values for the test of normality	0.288	0.745					
Visit 2_ Hemoglobin Hb				Wilcoxon rank sum test	(-5 , 2)	W = 7794	0.474
Mean (SD)	144.73 (13.90)	146.07 (12.77)	145.35 (13.37)				
Median (Q1, Q3)	146.00 (137.00, 154.00)	145.00 (138.75, 154.00)	146.00 (137.00, 154.00)				
Min - Max	107.00 - 175.00	99.00 - 171.00	99.00 - 175.00				
Missing	14	29	43				
Average rank	125.891	132.55	129				
P-values for the test of normality	0.692	0.035					

variable	Test group (N=151)	Control group (N=149)	Total (N=300)	method of calibration	95%CI	statistics	P price
Visit 3_ Hemoglobin Hb				t-test	(-2.519 , 4.564)	t = 0.57	0.570
Mean (SD)	146.84 (14.29)	145.82 (12.97)	146.36 (13.67)				
Median (Q1, Q3)	147.50 (134.75, 157.25)	148.00 (137.00, 156.00)	148.00 (136.00, 156.00)				
Min - Max	117.00 - 178.00	101.00 - 174.00	101.00 - 178.00				
Missing	27	40	67				
P-values for the test of normality	0.067	0.076					
Visit 4_ Hb Hb				t-test	(-5.027 , 2.333)	t = -0.72	0.472
Mean (SD)	146.74 (14.77)	148.09 (15.19)	147.40 (14.96)				
Median (Q1, Q3)	148.00 (136.00, 156.00)	149.00 (139.00, 156.75)	149.00 (138.00, 156.00)				
Min - Max	109.00 - 179.00	95.00 - 183.00	95.00 - 183.00				
Missing	20	23	43				
P-values for the test of normality	0.294	0.174					

(2) Baseline and each view group

variable	group	base line	final result	method of calibration	95%CI	statistics	P price
Visit 2_ Hemoglobin Hb	Control group (N=149)			Wilcoxon signed rank test	(-1.5 , 1)	T = 3197	0.821
	Mean (SD)	145.37 (13.90)	146.07 (12.77)				
	Median (Q1, Q3)	145.00 (136.50, 155.00)	145.00 (138.75, 154.00)				
	Min - Max	100.00 - 183.00	99.00 - 171.00				
	Missing	2	29				
	Test group (N=151)			Wilcoxon signed rank test	(-1.5 , 1.5)	T = 4111.5	0.97
	Mean (SD)	144.57 (13.45)	144.73 (13.90)				
	Median (Q1, Q3)	145.00 (135.00, 154.00)	146.00 (137.00, 154.00)				
	Min - Max	104.00 - 176.00	107.00 - 175.00				
	Missing	1	14				
Visit 3_ Hemoglobin Hb	Control group (N=149)			Wilcoxon signed rank test	(-3 , 0)	T = 1893	0.021
	Mean (SD)	145.37 (13.90)	145.82 (12.97)				
	Median (Q1, Q3)	145.00 (136.50, 155.00)	148.00 (137.00, 156.00)				

variable	group	base line	final result	method of calibration	95%CI	statistics	P price
	Min - Max	100.00 - 183.00	101.00 - 174.00				
	Missing	2	40				
	Test group (N=151)			Wilcoxon signed rank test	(-4 , -0.5)	T = 2501	0.02
	Mean (SD)	144.57 (13.45)	146.84 (14.29)				
	Median (Q1, Q3)	145.00 (135.00, 154.00)	147.50 (134.75, 157.25)				
	Min - Max	104.00 - 176.00	117.00 - 178.00				
	Missing	1	27				
Visit 4_ Hb Hb	Control group (N=149)			Wilcoxon signed rank test	(-3.5 , -0.5)	T = 2701.5	0.007
	Mean (SD)	145.37 (13.90)	148.09 (15.19)				
	Median (Q1, Q3)	145.00 (136.50, 155.00)	149.00 (139.00, 156.75)				
	Min - Max	100.00 - 183.00	95.00 - 183.00				
	Missing	2	23				
	Test group (N=151)			Wilcoxon signed rank test	(-4 , -0.5)	T = 3005	0.008
	Mean (SD)	144.57 (13.45)	146.74 (14.77)				

variable	group	base line	final result	method of calibration	95%CI	statistics	P price
	Median (Q1, Q3)	145.00 (135.00, 154.00)	148.00 (136.00, 156.00)				
	Min - Max	104.00 - 176.00	109.00 - 179.00				
	Missing	1	20				

1.3 White blood cell count, WBC

(1) Comparison between the groups of the visiting viewpoints

variable	Test group (N=151)	Control group (N=149)	Total (N=300)	method of calibration	95%CI	statistics	P price
Visit 1_ White blood cell count WBC				Wilcoxon rank sum test	(-0.3 , 0.36)	W = 11173.5	0.841
Mean (SD)	6.27 (1.67)	6.20 (1.57)	6.24 (1.62)				
Median (Q1, Q3)	6.05 (5.12, 7.18)	6.10 (5.14, 6.96)	6.09 (5.13, 6.98)				
Min - Max	3.20 - 12.42	3.12 - 14.28	3.12 - 14.28				
Missing	1	2	3				
Average rank	149.99	147.99	149				
P-values for the test of normality	< 0.001	< 0.001					
Visit 2_ WBC count WBC				Wilcoxon rank sum test	(-0.23 , 0.47)	W = 8601.5	0.522
Mean (SD)	6.30 (1.52)	6.20 (1.53)	6.25 (1.52)				
Median (Q1, Q3)	6.14 (5.28, 7.17)	6.02 (5.10, 6.97)	6.06 (5.20, 7.10)				
Min - Max	3.10 - 11.44	3.53 - 11.34	3.10 - 11.44				
Missing	14	29	43				
Average rank	131.785	125.821	129				

variable	Test group (N=151)	Control group (N=149)	Total (N=300)	method of calibration	95%CI	statistics	P price
P-values for the test of normality	< 0.001	< 0.001					
Visit 3_ White BC count WBC				Wilcoxon rank sum test	(-0.35 , 0.44)	W = 6888	0.801
Mean (SD)	6.45 (1.82)	6.34 (1.43)	6.40 (1.65)				
Median (Q1, Q3)	6.25 (5.19, 7.42)	6.06 (5.37, 7.19)	6.19 (5.28, 7.26)				
Min - Max	3.54 - 17.10	3.65 - 10.30	3.54 - 17.10				
Missing	27	40	67				
Average rank	118.048	115.807	117				
P-values for the test of normality	< 0.001	0.001					
Visit 4_ White BC count WBC				Wilcoxon rank sum test	(-0.41 , 0.33)	W = 8145.5	0.857
Mean (SD)	6.26 (1.55)	6.45 (1.85)	6.35 (1.70)				
Median (Q1, Q3)	6.18 (5.12, 7.17)	6.03 (5.33, 7.02)	6.06 (5.28, 7.10)				
Min - Max	3.20 - 11.97	2.61 - 14.57	2.61 - 14.57				
Missing	20	23	43				

variable	Test group (N=151)	Control group (N=149)	Total (N=300)	method of calibration	95%CI	statistics	P price
Average rank	128.179	129.853	129				
P-values for the test of normality	0.016	< 0.001					

(2) Baseline and each view group

variable	group	base line	final result	method of calibration	95%CI	statistics	P price
Visit 2_ WBC count WBC	Control group (N=149)			Wilcoxon signed rank test	(-0.225 , 0.165)	T = 3296	0.673
	Mean (SD)	6.20 (1.57)	6.20 (1.53)				
	Median (Q1, Q3)	6.10 (5.14, 6.96)	6.02 (5.10, 6.97)				
	Min - Max	3.12 - 14.28	3.53 - 11.34				
	Missing	2	29				
	Test group (N=151)			Wilcoxon signed rank test	(-0.185 , 0.19)	T = 4443	0.978
	Mean (SD)	6.27 (1.67)	6.30 (1.52)				
	Median (Q1, Q3)	6.05 (5.12, 7.18)	6.14 (5.28, 7.17)				

variable	group	base line	final result	method of calibration	95%CI	statistics	P price
	Min - Max	3.20 - 12.42	3.10 - 11.44				
	Missing	1	14				
Visit 3_ White BC count WBC	Control group (N=149)			Paired t-test	(-0.404 , 0.036)	t = -1.658	0.1
	Mean (SD)	6.20 (1.57)	6.34 (1.43)				
	Median (Q1, Q3)	6.10 (5.14, 6.96)	6.06 (5.37, 7.19)				
	Min - Max	3.12 - 14.28	3.65 - 10.30				
	Missing	2	40				
	Test group (N=151)			Wilcoxon signed rank test	(-0.285 , 0.055)	T = 3305	0.2
	Mean (SD)	6.27 (1.67)	6.45 (1.82)				
	Median (Q1, Q3)	6.05 (5.12, 7.18)	6.25 (5.19, 7.42)				
	Min - Max	3.20 - 12.42	3.54 - 17.10				
	Missing	1	27				
Visit 4_ White BC count WBC	Control group (N=149)			Wilcoxon signed rank test	(-0.37 , 0.075)	T = 3402.5	0.188
	Mean (SD)	6.20 (1.57)	6.45 (1.85)				

variable	group	base line	final result	method of calibration	95%CI	statistics	P price
	Median (Q1, Q3)	6.10 (5.14, 6.96)	6.03 (5.33, 7.02)				
	Min - Max	3.12 - 14.28	2.61 - 14.57				
	Missing	2	23				
	Test group (N=151)			Paired t-test	(-0.11 , 0.298)	t = 0.915	0.362
	Mean (SD)	6.27 (1.67)	6.26 (1.55)				
	Median (Q1, Q3)	6.05 (5.12, 7.18)	6.18 (5.12, 7.17)				
	Min - Max	3.20 - 12.42	3.20 - 11.97				
	Missing	1	20				

1.4 Platelet count, PLT

(1) Comparison between the groups of the visiting viewpoints

variable	Test group (N=151)	Control group (N=149)	Total (N=300)	method of calibration	95%CI	statistics	P price
Visit 1_ Platelet Count PLT				Wilcoxon rank sum test	(-5 , 20)	W = 11949	0.212
Mean (SD)	208.90 (53.98)	203.23 (54.51)	206.09 (54.23)				
Median (Q1, Q3)	206.00 (176.25, 240.00)	195.00 (165.00, 237.50)	203.00 (169.00, 240.00)				
Min - Max	21.00 - 379.00	88.00 - 455.00	21.00 - 455.00				
Missing	1	2	3				
Average rank	155.16	142.714	149				
P-values for the test of normality	0.347	< 0.001					
Visit 2_ Platelet Count PLT				t-test	(-3.15 , 22.293)	t = 1.48	0.140
Mean (SD)	211.79 (50.93)	202.22 (52.50)	207.32 (51.79)				
Median (Q1, Q3)	213.00 (180.00, 249.00)	201.50 (162.50, 239.50)	206.00 (173.00, 245.00)				
Min - Max	35.00 - 364.00	79.00 - 334.00	35.00 - 364.00				
Missing	14	29	43				
P-values for the test of normality	0.457	0.382					

variable	Test group (N=151)	Control group (N=149)	Total (N=300)	method of calibration	95%CI	statistics	P price
Visit 3_ Platelet count PLT				t-test	(-4.066 , 22.978)	t = 1.38	0.170
Mean (SD)	214.22 (51.05)	204.76 (53.62)	209.79 (52.37)				
Median (Q1, Q3)	211.00 (184.50, 250.00)	204.00 (163.00, 248.00)	211.00 (177.00, 250.00)				
Min - Max	25.00 - 339.00	83.00 - 313.00	25.00 - 339.00				
Missing	27	40	67				
P-values for the test of normality	0.106	0.15					
Visit 4_ Platelet count PLT				Wilcoxon rank sum test	(-8 , 19)	W = 8749	0.406
Mean (SD)	209.73 (53.19)	204.56 (54.06)	207.19 (53.58)				
Median (Q1, Q3)	203.00 (174.00, 243.50)	197.50 (162.00, 244.00)	200.00 (168.00, 244.00)				
Min - Max	58.00 - 356.00	90.00 - 334.00	58.00 - 356.00				
Missing	20	23	43				
Average rank	132.786	125.063	129				
P-values for the test of normality	0.065	0.032					

(2) Baseline and each view group

variable	group	base line	final result	method of calibration	95%CI	statistics	P price
Visit 2_ Platelet Count PLT	Control group (N=149)			Wilcoxon signed rank test	(-6 , 2.5)	T = 3109	0.435
	Mean (SD)	203.23 (54.51)	202.22 (52.50)				
	Median (Q1, Q3)	195.00 (165.00, 237.50)	201.50 (162.50, 239.50)				
	Min - Max	88.00 - 455.00	79.00 - 334.00				
	Missing	2	29				
	Test group (N=151)			Wilcoxon signed rank test	(-7 , 1)	T = 3849.5	0.174
	Mean (SD)	208.90 (53.98)	211.79 (50.93)				
	Median (Q1, Q3)	206.00 (176.25, 240.00)	213.00 (180.00, 249.00)				
	Min - Max	21.00 - 379.00	35.00 - 364.00				
	Missing	1	14				
Visit 3_ Platelet count PLT	Control group (N=149)			Wilcoxon signed rank test	(-10 , 0.5)	T = 2163	0.066
	Mean (SD)	203.23 (54.51)	204.76 (53.62)				
	Median (Q1, Q3)	195.00 (165.00, 237.50)	204.00 (163.00, 248.00)				

variable	group	base line	final result	method of calibration	95%CI	statistics	P price
	Min - Max	88.00 - 455.00	83.00 - 313.00				
	Missing	2	40				
	Test group (N=151)			Wilcoxon signed rank test	(-6.5 , 3.5)	T = 3553.5	0.614
	Mean (SD)	208.90 (53.98)	214.22 (51.05)				
	Median (Q1, Q3)	206.00 (176.25, 240.00)	211.00 (184.50, 250.00)				
	Min - Max	21.00 - 379.00	25.00 - 339.00				
	Missing	1	27				
Visit 4_ Platelet count PLT	Control group (N=149)			Wilcoxon signed rank test	(-6.5 , 4)	T = 3730	0.719
	Mean (SD)	203.23 (54.51)	204.56 (54.06)				
	Median (Q1, Q3)	195.00 (165.00, 237.50)	197.50 (162.00, 244.00)				
	Min - Max	88.00 - 455.00	90.00 - 334.00				
	Missing	2	23				
	Test group (N=151)			Wilcoxon signed rank test	(-6 , 6.5)	T = 4027	0.95
	Mean (SD)	208.90 (53.98)	209.73 (53.19)				

variable	group	base line	final result	method of calibration	95%CI	statistics	P price
	Median (Q1, Q3)	206.00 (176.25, 240.00)	203.00 (174.00, 243.50)				
	Min - Max	21.00 - 379.00	58.00 - 356.00				
	Missing	1	20				

2. Liver work

2.1 albumin ALB

(1) Comparison between the groups of the visiting viewpoints

variable	Test group (N=151)	Control group (N=149)	Total (N=300)	method of calibration	95%CI	statistics	P price
Visit 1_ Albumin-ALB				Wilcoxon rank sum test	(-1.2 , 0.3)	W = 9558.5	0.216
Mean (SD)	45.07 (3.13)	45.34 (3.11)	45.20 (3.12)				
Median (Q1, Q3)	44.50 (42.90, 47.14)	45.30 (43.42, 47.10)	45.10 (43.00, 47.10)				
Min - Max	38.50 - 56.50	35.40 - 53.30	35.40 - 56.50				
Missing	4	7	11				
Average rank	139.024	151.187	145				
P-values for the test of normality	0.007	0.718					
Visit 2_ Albumin-ALB				Wilcoxon rank sum test	(-1.4 , 0.1)	W = 7439	0.103
Mean (SD)	44.97 (3.06)	45.48 (3.41)	45.21 (3.24)				
Median (Q1, Q3)	44.80 (42.80, 47.00)	45.70 (43.65, 47.49)	45.40 (43.10, 47.23)				
Min - Max	39.00 - 53.50	31.10 - 54.40	31.10 - 54.40				
Missing	14	26	40				
Average rank	123.299	138.52	130.5				

variable	Test group (N=151)	Control group (N=149)	Total (N=300)	method of calibration	95%CI	statistics	P price
P-values for the test of normality	0.17	0.005					
Visit 3_ Albumin ALB				t-test	(-0.929 , 0.776)	t = -0.18	0.859
Mean (SD)	45.12 (3.27)	45.20 (3.32)	45.16 (3.29)				
Median (Q1, Q3)	44.80 (42.80, 47.30)	45.40 (43.20, 47.70)	45.02 (43.10, 47.60)				
Min - Max	36.50 - 53.70	35.00 - 53.70	35.00 - 53.70				
Missing	27	40	67				
P-values for the test of normality	0.444	0.742					
Visit 4_ Albumin ALB				Wilcoxon rank sum test	(-1.2 , 0.3)	W = 7635	0.256
Mean (SD)	44.57 (3.32)	44.89 (2.90)	44.73 (3.12)				
Median (Q1, Q3)	44.50 (42.30, 46.23)	44.85 (42.73, 47.25)	44.70 (42.60, 46.70)				
Min - Max	36.50 - 57.90	38.40 - 51.10	36.50 - 57.90				
Missing	19	23	42				
Average rank	124.341	134.905	129.5				

variable	Test group (N=151)	Control group (N=149)	Total (N=300)	method of calibration	95%CI	statistics	P price
P-values for the test of normality	0.001	0.287					

(2) Baseline and each view group

variable	group	base line	final result	method of calibration	95%CI	statistics	P price
Visit 2_ Albumin-ALB	Control group (N=149)			Wilcoxon signed rank test	(-0.8 , 0.1)	T = 2935.5	0.123
	Mean (SD)	45.34 (3.11)	45.48 (3.41)				
	Median (Q1, Q3)	45.30 (43.42, 47.10)	45.70 (43.65, 47.49)				
	Min - Max	35.40 - 53.30	31.10 - 54.40				
	Missing	7	26				
	Test group (N=151)			Wilcoxon signed rank test	(-0.5 , 0.49)	T = 4141.5	0.975
	Mean (SD)	45.07 (3.13)	44.97 (3.06)				
	Median (Q1, Q3)	44.50 (42.90, 47.14)	44.80 (42.80, 47.00)				
	Min - Max	38.50 - 56.50	39.00 - 53.50				
	Missing	4	14				

variable	group	base line	final result	method of calibration	95%CI	statistics	P price
Visit 3_ Albumin ALB	Control group (N=149)			Wilcoxon signed rank test	(-0.85 , 0.3)	T = 2364.5	0.303
	Mean (SD)	45.34 (3.11)	45.20 (3.32)				
	Median (Q1, Q3)	45.30 (43.42, 47.10)	45.40 (43.20, 47.70)				
	Min - Max	35.40 - 53.30	35.00 - 53.70				
	Missing	7	40				
	Test group (N=151)			Wilcoxon signed rank test	(-0.7 , 0.32)	T = 3266.5	0.513
	Mean (SD)	45.07 (3.13)	45.12 (3.27)				
	Median (Q1, Q3)	44.50 (42.90, 47.14)	44.80 (42.80, 47.30)				
	Min - Max	38.50 - 56.50	36.50 - 53.70				
	Missing	4	27				
Visit 4_ Albumin ALB	Control group (N=149)			Paired t-test	(0.043 , 1.187)	t = 2.127	0.035
	Mean (SD)	45.34 (3.11)	44.89 (2.90)				
	Median (Q1, Q3)	45.30 (43.42, 47.10)	44.85 (42.73, 47.25)				

variable	group	base line	final result	method of calibration	95%CI	statistics	P price
	Min - Max	35.40 - 53.30	38.40 - 51.10				
	Missing	7	23				
	Test group (N=151)			Paired t-test	(-0.057 , 1.074)	t = 1.778	0.078
	Mean (SD)	45.07 (3.13)	44.57 (3.32)				
	Median (Q1, Q3)	44.50 (42.90, 47.14)	44.50 (42.30, 46.23)				
	Min - Max	38.50 - 56.50	36.50 - 57.90				
	Missing	4	19				

2.2 ALT ALT

(1) Comparison between the groups of the visiting viewpoints

variable	Test group (N=151)	Control group (N=149)	Total (N=300)	method of calibration	95%CI	statistics	P price
Visit 1_ ALT ALT				Wilcoxon rank sum test	(-2 , 2)	W = 11133	0.884
Mean (SD)	28.57 (21.81)	31.57 (69.63)	30.05 (51.31)				
Median (Q1, Q3)	22.00 (17.00, 33.00)	23.00 (17.00, 30.10)	22.00 (17.00, 32.00)				
Min - Max	8.00 - 185.00	9.00 - 856.00	8.00 - 856.00				
Missing	1	2	3				
Average rank	149.72	148.265	149				
P-values for the test of normality	< 0.001	< 0.001					
Visit 2_ ALT ALT				Wilcoxon rank sum test	(-3 , 2)	W = 8159.5	0.583
Mean (SD)	33.40 (56.25)	28.06 (16.42)	30.86 (42.30)				
Median (Q1, Q3)	23.00 (17.00, 33.50)	24.50 (18.00, 34.20)	23.00 (18.00, 34.00)				
Min - Max	7.00 - 638.10	6.00 - 133.00	6.00 - 638.10				
Missing	14	25	39				
Average rank	128.558	133.698	131				

variable	Test group (N=151)	Control group (N=149)	Total (N=300)	method of calibration	95%CI	statistics	P price
P-values for the test of normality	< 0.001	< 0.001					
Visit 3_ ALT ALT				Wilcoxon rank sum test	(-3 , 3)	W = 6790	0.951
Mean (SD)	30.01 (21.13)	27.47 (13.52)	28.82 (17.98)				
Median (Q1, Q3)	25.00 (19.00, 33.00)	26.00 (17.00, 35.00)	25.00 (18.00, 33.20)				
Min - Max	7.00 - 136.00	7.00 - 80.00	7.00 - 136.00				
Missing	27	40	67				
Average rank	117.258	116.706	117				
P-values for the test of normality	< 0.001	< 0.001					
Visit 4_ GT ALT				Wilcoxon rank sum test	(-4 , 1)	W = 7773	0.365
Mean (SD)	25.99 (14.05)	27.31 (14.81)	26.64 (14.42)				
Median (Q1, Q3)	22.00 (16.00, 30.25)	24.00 (17.00, 33.75)	23.00 (17.00, 32.75)				
Min - Max	7.00 - 98.00	4.00 - 104.20	4.00 - 104.20				
Missing	19	23	42				

variable	Test group (N=151)	Control group (N=149)	Total (N=300)	method of calibration	95%CI	statistics	P price
Average rank	125.386	133.81	129.5				
P-values for the test of normality	< 0.001	< 0.001					

(2) Baseline and each view group

variable	group	base line	final result	method of calibration	95%CI	statistics	P price
Visit 2_ ALT ALT	Control group (N=149)			Wilcoxon signed rank test	(-2 ， 1)	T = 3210.5	0.616
	Mean (SD)	31.57 (69.63)	28.06 (16.42)				
	Median (Q1, Q3)	23.00 (17.00, 30.10)	24.50 (18.00, 34.20)				
	Min - Max	9.00 - 856.00	6.00 - 133.00				
	Missing	2	25				
	Test group (N=151)			Wilcoxon signed rank test	(-2 ， 1)	T = 4139	0.673
	Mean (SD)	28.57 (21.81)	33.40 (56.25)				
	Median (Q1, Q3)	22.00 (17.00, 33.00)	23.00 (17.00, 33.50)				

variable	group	base line	final result	method of calibration	95%CI	statistics	P price
	Min - Max	8.00 - 185.00	7.00 - 638.10				
	Missing	1	14				
Visit 3_ ALT ALT	Control group (N=149)			Wilcoxon signed rank test	(-4.15 , -0.15)	T = 2103	0.03
	Mean (SD)	31.57 (69.63)	27.47 (13.52)				
	Median (Q1, Q3)	23.00 (17.00, 30.10)	26.00 (17.00, 35.00)				
	Min - Max	9.00 - 856.00	7.00 - 80.00				
	Missing	2	40				
	Test group (N=151)			Wilcoxon signed rank test	(-3.5 , 1)	T = 3167.5	0.286
	Mean (SD)	28.57 (21.81)	30.01 (21.13)				
	Median (Q1, Q3)	22.00 (17.00, 33.00)	25.00 (19.00, 33.00)				
	Min - Max	8.00 - 185.00	7.00 - 136.00				
	Missing	1	27				
Visit 4_ GT ALT	Control group (N=149)			Wilcoxon signed rank test	(-2.05 , 1.5)	T = 3400	0.768
	Mean (SD)	31.57 (69.63)	27.31 (14.81)				

variable	group	base line	final result	method of calibration	95%CI	statistics	P price
	Median (Q1, Q3)	23.00 (17.00, 30.10)	24.00 (17.00, 33.75)				
	Min - Max	9.00 - 856.00	4.00 - 104.20				
	Missing	2	23				
	Test group (N=151)			Wilcoxon signed rank test	(-1 , 3)	T = 4461	0.34
	Mean (SD)	28.57 (21.81)	25.99 (14.05)				
	Median (Q1, Q3)	22.00 (17.00, 33.00)	22.00 (16.00, 30.25)				
	Min - Max	8.00 - 185.00	7.00 - 98.00				
	Missing	1	19				

2.3 Valley Grass transaminase AST

(1) Comparison between the groups of the visiting viewpoints

variable	Test group (N=151)	Control group (N=149)	Total (N=300)	method of calibration	95%CI	statistics	P price
Visit 1_ transaminase AST				Wilcoxon rank sum test	(-2 , 1)	W = 10823.5	0.712
Mean (SD)	25.04 (10.35)	27.52 (41.32)	26.26 (29.92)				
Median (Q1, Q3)	22.00 (19.00, 28.00)	23.00 (18.15, 28.00)	22.00 (18.48, 28.00)				
Min - Max	10.00 - 76.70	10.00 - 516.00	10.00 - 516.00				
Missing	0	2	2				
Average rank	147.679	151.371	149.5				
P-values for the test of normality	< 0.001	< 0.001					
Visit 2_ transaminase AST				Wilcoxon rank sum test	(-2 , 1)	W = 8160	0.584
Mean (SD)	27.54 (37.00)	24.81 (10.36)	26.24 (27.72)				
Median (Q1, Q3)	23.00 (19.00, 29.00)	23.00 (19.00, 28.00)	23.00 (19.00, 28.00)				
Min - Max	12.00 - 440.50	11.00 - 94.00	11.00 - 440.50				
Missing	14	25	39				
Average rank	128.562	133.694	131				

variable	Test group (N=151)	Control group (N=149)	Total (N=300)	method of calibration	95%CI	statistics	P price
P-values for the test of normality	< 0.001	< 0.001					
Visit 3_ transaminase AST				Wilcoxon rank sum test	(-2 , 2)	W = 6727	0.953
Mean (SD)	25.61 (10.82)	24.89 (8.44)	25.27 (9.76)				
Median (Q1, Q3)	23.00 (19.00, 27.00)	24.00 (18.00, 30.00)	24.00 (19.00, 29.00)				
Min - Max	11.00 - 80.60	11.00 - 52.00	11.00 - 80.60				
Missing	27	40	67				
Average rank	116.75	117.284	117				
P-values for the test of normality	< 0.001	0.001					
Visit 4_ transaminase AST				Wilcoxon rank sum test	(-3 , 1)	W = 7531.5	0.190
Mean (SD)	23.77 (7.68)	25.01 (8.23)	24.38 (7.96)				
Median (Q1, Q3)	22.50 (18.30, 27.00)	24.00 (19.00, 29.15)	23.00 (19.00, 28.00)				
Min - Max	11.00 - 49.00	13.00 - 55.90	11.00 - 55.90				
Missing	19	23	42				

variable	Test group (N=151)	Control group (N=149)	Total (N=300)	method of calibration	95%CI	statistics	P price
Average rank	123.557	135.726	129.5				
P-values for the test of normality	< 0.001	< 0.001					

(2) Baseline and each view group

variable	group	base line	final result	method of calibration	95%CI	statistics	P price
Visit 2_ transaminase AST	Control group (N=149)			Wilcoxon signed rank test	(-1 , 1.5)	T = 3452.5	0.621
	Mean (SD)	27.52 (41.32)	24.81 (10.36)				
	Median (Q1, Q3)	23.00 (18.15, 28.00)	23.00 (19.00, 28.00)				
	Min - Max	10.00 - 516.00	11.00 - 94.00				
	Missing	2	25				
	Test group (N=151)			Wilcoxon signed rank test	(-1 , 1.2)	T = 4349.5	0.713
	Mean (SD)	25.04 (10.35)	27.54 (37.00)				
	Median (Q1, Q3)	22.00 (19.00, 28.00)	23.00 (19.00, 29.00)				

variable	group	base line	final result	method of calibration	95%CI	statistics	P price
	Min - Max	10.00 - 76.70	12.00 - 440.50				
	Missing	0	14				
Visit 3_ transaminase AST	Control group (N=149)			Wilcoxon signed rank test	(-3.35 , 0)	T = 1715	0.072
	Mean (SD)	27.52 (41.32)	24.89 (8.44)				
	Median (Q1, Q3)	23.00 (18.15, 28.00)	24.00 (18.00, 30.00)				
	Min - Max	10.00 - 516.00	11.00 - 52.00				
	Missing	2	40				
	Test group (N=151)			Wilcoxon signed rank test	(-1.5 , 1)	T = 3377.5	0.61
	Mean (SD)	25.04 (10.35)	25.61 (10.82)				
	Median (Q1, Q3)	22.00 (19.00, 28.00)	23.00 (19.00, 27.00)				
	Min - Max	10.00 - 76.70	11.00 - 80.60				
	Missing	0	27				
Visit 4_ transaminase AST	Control group (N=149)			Wilcoxon signed rank test	(-2 , 0.5)	T = 3099.5	0.27
	Mean (SD)	27.52 (41.32)	25.01 (8.23)				

variable	group	base line	final result	method of calibration	95%CI	statistics	P price
	Median (Q1, Q3)	23.00 (18.15, 28.00)	24.00 (19.00, 29.15)				
	Min - Max	10.00 - 516.00	13.00 - 55.90				
	Missing	2	23				
	Test group (N=151)			Wilcoxon signed rank test	(-1 , 1.55)	T = 3821.5	0.735
	Mean (SD)	25.04 (10.35)	23.77 (7.68)				
	Median (Q1, Q3)	22.00 (19.00, 28.00)	22.50 (18.30, 27.00)				
	Min - Max	10.00 - 76.70	11.00 - 49.00				
	Missing	0	19				

2.4 Total bilirubin TB

(1) Comparison between the groups of the visiting viewpoints

variable	Test group (N=151)	Control group (N=149)	Total (N=300)	method of calibration	95%CI	statistics	P price
Visit 1_ Total bilirubin TB				Wilcoxon rank sum test	(-0.7 , 1.7)	W = 11676	0.438
Mean (SD)	15.32 (6.84)	14.71 (6.99)	15.02 (6.91)				
Median (Q1, Q3)	13.80 (10.85, 17.80)	13.40 (10.45, 17.35)	13.70 (10.50, 17.70)				
Min - Max	6.10 - 45.07	2.30 - 55.30	2.30 - 55.30				
Missing	0	2	2				
Average rank	153.325	145.571	149.5				
P-values for the test of normality	< 0.001	< 0.001					
Visit 2_ Total bilirubin TB				Wilcoxon rank sum test	(-2.3 , 0.3)	W = 7540	0.117
Mean (SD)	14.49 (6.29)	15.44 (6.38)	14.94 (6.34)				
Median (Q1, Q3)	13.00 (10.10, 17.10)	14.10 (10.90, 18.16)	13.60 (10.50, 17.80)				
Min - Max	5.20 - 34.00	6.20 - 39.85	5.20 - 39.85				
Missing	14	25	39				
Average rank	124.036	138.694	131				

variable	Test group (N=151)	Control group (N=149)	Total (N=300)	method of calibration	95%CI	statistics	P price
P-values for the test of normality	< 0.001	< 0.001					
Visit 3_ Total bilirubin TB				Wilcoxon rank sum test	(-1.3 , 1.5)	W = 6857	0.848
Mean (SD)	14.75 (5.93)	14.83 (6.41)	14.79 (6.14)				
Median (Q1, Q3)	14.10 (10.97, 17.60)	13.80 (10.20, 17.70)	13.90 (10.70, 17.60)				
Min - Max	4.50 - 37.50	4.90 - 40.08	4.50 - 40.08				
Missing	27	40	67				
Average rank	117.798	116.092	117				
P-values for the test of normality	< 0.001	< 0.001					
Visit 4_ Total bilirubin TB				Wilcoxon rank sum test	(-1.9 , 0.7)	W = 7883	0.408
Mean (SD)	14.23 (5.46)	15.18 (6.61)	14.69 (6.06)				
Median (Q1, Q3)	13.10 (10.80, 17.00)	13.50 (10.80, 17.82)	13.20 (10.80, 17.41)				
Min - Max	3.30 - 33.70	5.30 - 38.70	3.30 - 38.70				
Missing	19	22	41				

variable	Test group (N=151)	Control group (N=149)	Total (N=300)	method of calibration	95%CI	statistics	P price
Average rank	126.22	133.929	130				
P-values for the test of normality	< 0.001	< 0.001					

(2) Baseline and each view group

variable	group	base line	final result	method of calibration	95%CI	statistics	P price
Visit 2_ Total bilirubin TB	Control group (N=149)			Paired t-test	(-1.141 , 0.112)	t = -1.625	0.107
	Mean (SD)	14.71 (6.99)	15.44 (6.38)				
	Median (Q1, Q3)	13.40 (10.45, 17.35)	14.10 (10.90, 18.16)				
	Min - Max	2.30 - 55.30	6.20 - 39.85				
	Missing	2	25				
	Test group (N=151)			Wilcoxon signed rank test	(-0.25 , 1.15)	T = 5108	0.194
	Mean (SD)	15.32 (6.84)	14.49 (6.29)				
	Median (Q1, Q3)	13.80 (10.85, 17.80)	13.00 (10.10, 17.10)				

variable	group	base line	final result	method of calibration	95%CI	statistics	P price
	Min - Max	6.10 - 45.07	5.20 - 34.00				
	Missing	0	14				
Visit 3_ Total bilirubin TB	Control group (N=149)			Paired t-test	(-0.524 , 0.885)	t = 0.509	0.612
	Mean (SD)	14.71 (6.99)	14.83 (6.41)				
	Median (Q1, Q3)	13.40 (10.45, 17.35)	13.80 (10.20, 17.70)				
	Min - Max	2.30 - 55.30	4.90 - 40.08				
	Missing	2	40				
	Test group (N=151)			Wilcoxon signed rank test	(-0.7 , 0.805)	T = 3914	0.924
	Mean (SD)	15.32 (6.84)	14.75 (5.93)				
	Median (Q1, Q3)	13.80 (10.85, 17.80)	14.10 (10.97, 17.60)				
	Min - Max	6.10 - 45.07	4.50 - 37.50				
	Missing	0	27				
Visit 4_ Total bilirubin TB	Control group (N=149)			Wilcoxon signed rank test	(-0.95 , 0.595)	T = 3742	0.631
	Mean (SD)	14.71 (6.99)	15.18 (6.61)				

variable	group	base line	final result	method of calibration	95%CI	statistics	P price
	Median (Q1, Q3)	13.40 (10.45, 17.35)	13.50 (10.80, 17.82)				
	Min - Max	2.30 - 55.30	5.30 - 38.70				
	Missing	2	22				
	Test group (N=151)			Wilcoxon signed rank test	(0 , 1.5)	T = 5243.5	0.052
	Mean (SD)	15.32 (6.84)	14.23 (5.46)				
	Median (Q1, Q3)	13.80 (10.85, 17.80)	13.10 (10.80, 17.00)				
	Min - Max	6.10 - 45.07	3.30 - 33.70				
	Missing	0	19				

2.5 Direct bilirubin DBIL

(1) Comparison between the groups of the visiting viewpoints

variable	Test group (N=151)	Control group (N=149)	Total (N=300)	method of calibration	95%CI	statistics	P price
Visit 1_ Direct bilirubin DBIL				Wilcoxon rank sum test	(-0.1 , 0.7)	W = 11494	0.168
Mean (SD)	4.56 (2.24)	4.30 (2.54)	4.43 (2.40)				
Median (Q1, Q3)	4.30 (3.01, 5.55)	3.90 (2.80, 5.20)	4.00 (2.92, 5.40)				
Min - Max	1.10 - 13.60	0.60 - 24.30	0.60 - 24.30				
Missing	4	6	10				
Average rank	152.19	138.622	145.5				
P-values for the test of normality	< 0.001	< 0.001					
Visit 2_ Direct bilirubin DBIL				Wilcoxon rank sum test	(-0.6 , 0.2)	W = 7819	0.372
Mean (SD)	4.29 (1.99)	4.49 (1.98)	4.39 (1.98)				
Median (Q1, Q3)	3.80 (3.10, 5.50)	4.15 (3.00, 5.75)	3.90 (3.00, 5.60)				
Min - Max	1.10 - 13.20	1.39 - 11.84	1.10 - 13.20				
Missing	14	27	41				

variable	Test group (N=151)	Control group (N=149)	Total (N=300)	method of calibration	95%CI	statistics	P price
Average rank	126.073	134.41	130				
P-values for the test of normality	< 0.001	< 0.001					
Visit 3_ Direct Bilirubin Reference Range Min				Wilcoxon rank sum test	(0 , 0)	W = 3421	0.602
Mean (SD)	0.01 (0.03)	0.01 (0.03)	0.01 (0.03)				
Median (Q1, Q3)	0.00 (0.00, 0.00)	0.00 (0.00, 0.00)	0.00 (0.00, 0.00)				
Min - Max	0.00 - 0.10	0.00 - 0.10	0.00 - 0.10				
Missing	62	70	132				
Average rank	83.438	85.696	84.5				
P-values for the test of normality	< 0.001	< 0.001					
Visit 4_ Direct bilirubin DBIL				Wilcoxon rank sum test	(-0.6 , 0.3)	W = 7922.5	0.512
Mean (SD)	4.24 (1.83)	4.38 (2.00)	4.31 (1.91)				
Median (Q1, Q3)	3.70 (2.90, 5.15)	4.20 (2.70, 5.68)	3.98 (2.80, 5.47)				

variable	Test group (N=151)	Control group (N=149)	Total (N=300)	method of calibration	95%CI	statistics	P price
Min - Max	1.50 - 10.50	0.20 - 11.30	0.20 - 11.30				
Missing	19	23	42				
Average rank	126.519	132.623	129.5				
P-values for the test of normality	< 0.001	0.003					

(2) Baseline and each view group

variable	group	base line	final result	method of calibration	95%CI	statistics	P price
Visit 2_ Direct bilirubin DBIL	Control group (N=149)			Wilcoxon signed rank test	(-0.45 , -0.05)	T = 2447	0.019
	Mean (SD)	4.30 (2.54)	4.49 (1.98)				
	Median (Q1, Q3)	3.90 (2.80, 5.20)	4.15 (3.00, 5.75)				
	Min - Max	0.60 - 24.30	1.39 - 11.84				
	Missing	6	27				
	Test group (N=151)			Wilcoxon signed rank test	(-0.005 , 0.45)	T = 4717	0.081
	Mean (SD)	4.56 (2.24)	4.29 (1.99)				

variable	group	base line	final result	method of calibration	95%CI	statistics	P price
	Median (Q1, Q3)	4.30 (3.01, 5.55)	3.80 (3.10, 5.50)				
	Min - Max	1.10 - 13.60	1.10 - 13.20				
	Missing	4	14				
Visit 3_ Direct Bilirubin Reference Range Min	Control group (N=149)			Wilcoxon signed rank test	(3.39 , 4.2)	T = 2926	< 0.001
	Mean (SD)	4.30 (2.54)	0.01 (0.03)				
	Median (Q1, Q3)	3.90 (2.80, 5.20)	0.00 (0.00, 0.00)				
	Min - Max	0.60 - 24.30	0.00 - 0.10				
	Missing	6	70				
	Test group (N=151)			Wilcoxon signed rank test	(3.95 , 4.75)	T = 3655	< 0.001
	Mean (SD)	4.56 (2.24)	0.01 (0.03)				
	Median (Q1, Q3)	4.30 (3.01, 5.55)	0.00 (0.00, 0.00)				
	Min - Max	1.10 - 13.60	0.00 - 0.10				
	Missing	4	62				

variable	group	base line	final result	method of calibration	95%CI	statistics	P price
Visit 4_ Direct bilirubin DBIL	Control group (N=149)			Wilcoxon signed rank test	(-0.4 , 0.18)	T = 3152.5	0.508
	Mean (SD)	4.30 (2.54)	4.38 (2.00)				
	Median (Q1, Q3)	3.90 (2.80, 5.20)	4.20 (2.70, 5.68)				
	Min - Max	0.60 - 24.30	0.20 - 11.30				
	Missing	6	23				
	Test group (N=151)			Wilcoxon signed rank test	(-0.05 , 0.45)	T = 4444.5	0.111
	Mean (SD)	4.56 (2.24)	4.24 (1.83)				
	Median (Q1, Q3)	4.30 (3.01, 5.55)	3.70 (2.90, 5.15)				
	Min - Max	1.10 - 13.60	1.50 - 10.50				
	Missing	4	19				

2.6 The indirect bilirubin IBIL

(1) Comparison between the groups of the visiting viewpoints

variable	Test group (N=151)	Control group (N=149)	Total (N=300)	method of calibration	95%CI	statistics	P price
Visit 1_ indirect bilirubin IBIL				Wilcoxon rank sum test	(-0.9 , 0.9)	W = 10543	0.964
Mean (SD)	10.55 (5.14)	10.45 (5.16)	10.50 (5.14)				
Median (Q1, Q3)	9.60 (7.00, 12.88)	9.70 (7.14, 11.75)	9.70 (7.03, 12.47)				
Min - Max	2.70 - 34.56	1.70 - 31.40	1.70 - 34.56				
Missing	4	6	10				
Average rank	145.721	145.273	145.5				
P-values for the test of normality	< 0.001	< 0.001					
Visit 2_ indirect bilirubin IBIL				Wilcoxon rank sum test	(-1.8 , 0.3)	W = 7482	0.146
Mean (SD)	10.23 (4.86)	11.01 (5.13)	10.59 (4.99)				
Median (Q1, Q3)	9.30 (6.90, 12.40)	9.60 (7.73, 13.15)	9.50 (7.05, 13.00)				
Min - Max	2.70 - 26.00	3.70 - 31.96	2.70 - 31.96				
Missing	14	27	41				
Average rank	123.613	137.172	130				

variable	Test group (N=151)	Control group (N=149)	Total (N=300)	method of calibration	95%CI	statistics	P price
P-values for the test of normality	< 0.001	< 0.001					
Visit 3_ Indirect bilirubin reference range minimum value				Wilcoxon rank sum test	(0 , 0)	W = 4559	0.738
Mean (SD)	1.57 (1.32)	1.64 (1.31)	1.60 (1.31)				
Median (Q1, Q3)	2.00 (0.00, 2.00)	2.00 (0.20, 2.70)	2.00 (0.05, 2.00)				
Min - Max	0.00 - 3.40	0.00 - 3.40	0.00 - 3.40				
Missing	48	58	106				
Average rank	96.262	98.901	97.5				
P-values for the test of normality	< 0.001	< 0.001					
Visit 4_ indirect bilirubin IBIL				Wilcoxon rank sum test	(-1.5 , 0.5)	W = 7715	0.369
Mean (SD)	9.95 (4.39)	10.77 (5.33)	10.35 (4.88)				
Median (Q1, Q3)	9.13 (6.85, 12.12)	9.30 (7.40, 13.30)	9.20 (7.00, 12.90)				

variable	Test group (N=151)	Control group (N=149)	Total (N=300)	method of calibration	95%CI	statistics	P price
Min - Max	2.00 - 26.20	2.60 - 30.30	2.00 - 30.30				
Missing	19	24	43				
Average rank	124.947	133.28	129				
P-values for the test of normality	< 0.001	< 0.001					

(2) Baseline and each view group

variable	group	base line	final result	method of calibration	95%CI	statistics	P price
Visit 2_ indirect bilirubin IBIL	Control group (N=149)			Paired t-test	(-0.803 , 0.142)	t = -1.384	0.169
	Mean (SD)	10.45 (5.16)	11.01 (5.13)				
	Median (Q1, Q3)	9.70 (7.14, 11.75)	9.60 (7.73, 13.15)				
	Min - Max	1.70 - 31.40	3.70 - 31.96				
	Missing	6	27				
	Test group (N=151)			Wilcoxon signed rank test	(-0.3 , 0.65)	T = 4544	0.409
	Mean (SD)	10.55 (5.14)	10.23 (4.86)				

variable	group	base line	final result	method of calibration	95%CI	statistics	P price
	Median (Q1, Q3)	9.60 (7.00, 12.88)	9.30 (6.90, 12.40)				
	Min - Max	2.70 - 34.56	2.70 - 26.00				
	Missing	4	14				
Visit 3_ Indirect bilirubin reference range minimum value	Control group (N=149)			Wilcoxon signed rank test	(7.25 , 8.92)	T = 3916	< 0.001
	Mean (SD)	10.45 (5.16)	1.64 (1.31)				
	Median (Q1, Q3)	9.70 (7.14, 11.75)	2.00 (0.20, 2.70)				
	Min - Max	1.70 - 31.40	0.00 - 3.40				
	Missing	6	58				
	Test group (N=151)			Wilcoxon signed rank test	(7.1 , 9.25)	T = 4950	< 0.001
	Mean (SD)	10.55 (5.14)	1.57 (1.32)				
	Median (Q1, Q3)	9.60 (7.00, 12.88)	2.00 (0.00, 2.00)				
	Min - Max	2.70 - 34.56	0.00 - 3.40				

variable	group	base line	final result	method of calibration	95%CI	statistics	P price
	Missing	4	48				
Visit 4_ indirect bilirubin IBIL	Control group (N=149)			Wilcoxon signed rank test	(-0.6 , 0.55)	T = 3459	0.891
	Mean (SD)	10.45 (5.16)	10.77 (5.33)				
	Median (Q1, Q3)	9.70 (7.14, 11.75)	9.30 (7.40, 13.30)				
	Min - Max	1.70 - 31.40	2.60 - 30.30				
	Missing	6	24				
	Test group (N=151)			Wilcoxon signed rank test	(0.05 , 1.2)	T = 4973	0.029
	Mean (SD)	10.55 (5.14)	9.95 (4.39)				
	Median (Q1, Q3)	9.60 (7.00, 12.88)	9.13 (6.85, 12.12)				
	Min - Max	2.70 - 34.56	2.00 - 26.20				
	Missing	4	19				

3 Renal work

3.1 Urea UREA

(1) Comparison between the groups of the visiting viewpoints

variable	Test group (N=151)	Control group (N=149)	Total (N=300)	method of calibration	95%CI	statistics	P price
Visit 1_ Urea UREA				Wilcoxon rank sum test	(-0.17 , 0.43)	W = 11782	0.358
Mean (SD)	5.43 (1.34)	5.41 (1.52)	5.42 (1.43)				
Median (Q1, Q3)	5.30 (4.61, 6.31)	5.18 (4.36, 6.22)	5.21 (4.46, 6.30)				
Min - Max	2.58 - 9.41	2.51 - 12.60	2.51 - 12.60				
Missing	0	2	2				
Average rank	154.026	144.85	149.5				
P-values for the test of normality	0.009	< 0.001					
Visit 2_ Urea UREA				Wilcoxon rank sum test	(-0.65 , 0.01)	W = 7231.5	0.059
Mean (SD)	5.25 (1.38)	5.57 (1.43)	5.40 (1.41)				
Median (Q1, Q3)	5.06 (4.33, 6.02)	5.50 (4.50, 6.19)	5.33 (4.34, 6.13)				
Min - Max	2.65 - 10.24	3.22 - 10.40	2.65 - 10.40				
Missing	16	25	41				
Average rank	121.567	139.181	130				

variable	Test group (N=151)	Control group (N=149)	Total (N=300)	method of calibration	95%CI	statistics	P price
P-values for the test of normality	< 0.001	< 0.001					
Visit 3_ Urea UREA				Wilcoxon rank sum test	(-0.6 , 0.15)	W = 6163	0.242
Mean (SD)	5.29 (1.62)	5.61 (2.11)	5.44 (1.87)				
Median (Q1, Q3)	5.06 (4.15, 6.11)	5.27 (4.50, 6.42)	5.15 (4.33, 6.20)				
Min - Max	2.44 - 10.76	2.24 - 21.55	2.24 - 21.55				
Missing	28	39	67				
Average rank	112.106	122.473	117				
P-values for the test of normality	< 0.001	< 0.001					
Visit 4_ Urea UREA				Wilcoxon rank sum test	(-0.52 , 0.11)	W = 7390	0.212
Mean (SD)	5.32 (1.49)	5.62 (1.92)	5.47 (1.72)				
Median (Q1, Q3)	5.07 (4.30, 6.02)	5.31 (4.60, 6.10)	5.22 (4.50, 6.06)				
Min - Max	1.75 - 10.30	2.63 - 19.14	1.75 - 19.14				
Missing	21	24	45				

variable	Test group (N=151)	Control group (N=149)	Total (N=300)	method of calibration	95%CI	statistics	P price
Average rank	122.346	133.88	128				
P-values for the test of normality	< 0.001	< 0.001					

(2) Baseline and each view group

variable	group	base line	final result	method of calibration	95%CI	statistics	P price
Visit 2_ Urea UREA	Control group (N=149)			Wilcoxon signed rank test	(-0.4 , 0.025)	T = 3063	0.079
	Mean (SD)	5.41 (1.52)	5.57 (1.43)				
	Median (Q1, Q3)	5.18 (4.36, 6.22)	5.50 (4.50, 6.19)				
	Min - Max	2.51 - 12.60	3.22 - 10.40				
	Missing	2	25				
	Test group (N=151)			Paired t-test	(-0.042 , 0.394)	t = 1.593	0.114
	Mean (SD)	5.43 (1.34)	5.25 (1.38)				
	Median (Q1, Q3)	5.30 (4.61, 6.31)	5.06 (4.33, 6.02)				

variable	group	base line	final result	method of calibration	95%CI	statistics	P price
	Min - Max	2.58 - 9.41	2.65 - 10.24				
	Missing	0	16				
Visit 3_ Urea UREA	Control group (N=149)			Wilcoxon signed rank test	(-0.335 , 0.145)	T = 2735	0.428
	Mean (SD)	5.41 (1.52)	5.61 (2.11)				
	Median (Q1, Q3)	5.18 (4.36, 6.22)	5.27 (4.50, 6.42)				
	Min - Max	2.51 - 12.60	2.24 - 21.55				
	Missing	2	39				
	Test group (N=151)			Paired t-test	(-0.075 , 0.488)	t = 1.45	0.15
	Mean (SD)	5.43 (1.34)	5.29 (1.62)				
	Median (Q1, Q3)	5.30 (4.61, 6.31)	5.06 (4.15, 6.11)				
	Min - Max	2.58 - 9.41	2.44 - 10.76				
	Missing	0	28				
Visit 4_ Urea UREA	Control group (N=149)			Wilcoxon signed rank test	(-0.395 , 0.095)	T = 3317.5	0.212
	Mean (SD)	5.41 (1.52)	5.62 (1.92)				

variable	group	base line	final result	method of calibration	95%CI	statistics	P price
	Median (Q1, Q3)	5.18 (4.36, 6.22)	5.31 (4.60, 6.10)				
	Min - Max	2.51 - 12.60	2.63 - 19.14				
	Missing	2	24				
	Test group (N=151)			Paired t-test	(-0.115 , 0.403)	t = 1.102	0.273
	Mean (SD)	5.43 (1.34)	5.32 (1.49)				
	Median (Q1, Q3)	5.30 (4.61, 6.31)	5.07 (4.30, 6.02)				
	Min - Max	2.58 - 9.41	1.75 - 10.30				
	Missing	0	21				

3.2 Creatinine, CREA

(1) Comparison between the groups of the visiting viewpoints

variable	Test group (N=151)	Control group (N=149)	Total (N=300)	method of calibration	95%CI	statistics	P price
Visit 1_ Creatinine CREA				Wilcoxon rank sum test	(-3 , 4.8)	W = 11413.5	0.672
Mean (SD)	71.97 (17.46)	72.09 (19.99)	72.03 (18.72)				
Median (Q1, Q3)	70.00 (61.00, 79.00)	68.00 (58.00, 83.40)	70.00 (59.30, 80.97)				
Min - Max	27.00 - 142.00	37.60 - 174.00	27.00 - 174.00				
Missing	0	2	2				
Average rank	151.586	147.357	149.5				
P-values for the test of normality	< 0.001	< 0.001					
Visit 2_ Creatinine CREA				Wilcoxon rank sum test	(-5 , 3.7)	W = 8168.5	0.739
Mean (SD)	72.02 (16.94)	73.01 (20.40)	72.49 (18.64)				
Median (Q1, Q3)	69.90 (61.30, 79.00)	72.25 (58.22, 83.25)	70.80 (60.25, 82.00)				
Min - Max	23.00 - 150.00	32.00 - 183.00	23.00 - 183.00				
Missing	16	25	41				
Average rank	128.507	131.625	130				

variable	Test group (N=151)	Control group (N=149)	Total (N=300)	method of calibration	95%CI	statistics	P price
P-values for the test of normality	< 0.001	< 0.001					
Visit 3_ Creatinine CREA				Wilcoxon rank sum test	(-5.6 , 3.1)	W = 6544	0.594
Mean (SD)	72.12 (16.44)	74.57 (24.48)	73.27 (20.60)				
Median (Q1, Q3)	70.00 (61.83, 78.85)	72.00 (59.68, 84.00)	70.25 (61.00, 81.22)				
Min - Max	41.50 - 149.00	42.80 - 240.00	41.50 - 240.00				
Missing	27	39	66				
Average rank	115.274	120.009	117.5				
P-values for the test of normality	< 0.001	< 0.001					
Visit 4_ Creatinine CREA				Wilcoxon rank sum test	(-5 , 3)	W = 7928.5	0.584
Mean (SD)	72.59 (16.98)	74.50 (22.65)	73.53 (19.97)				
Median (Q1, Q3)	71.00 (62.00, 81.00)	72.90 (60.50, 86.00)	72.00 (61.00, 82.80)				
Min - Max	26.00 - 151.00	39.00 - 240.00	26.00 - 240.00				
Missing	21	22	43				

variable	Test group (N=151)	Control group (N=149)	Total (N=300)	method of calibration	95%CI	statistics	P price
Average rank	126.488	131.571	129				
P-values for the test of normality	< 0.001	< 0.001					

(2) Baseline and each view group

variable	group	base line	final result	method of calibration	95%CI	statistics	P price
Visit 2_ Creatinine CREA	Control group (N=149)			Wilcoxon signed rank test	(-2.9 , 0)	T = 2834.5	0.051
	Mean (SD)	72.09 (19.99)	73.01 (20.40)				
	Median (Q1, Q3)	68.00 (58.00, 83.40)	72.25 (58.22, 83.25)				
	Min - Max	37.60 - 174.00	32.00 - 183.00				
	Missing	2	25				
	Test group (N=151)			Wilcoxon signed rank test	(-1.5 , 1.5)	T = 4334	0.901
	Mean (SD)	71.97 (17.46)	72.02 (16.94)				
	Median (Q1, Q3)	70.00 (61.00, 79.00)	69.90 (61.30, 79.00)				

variable	group	base line	final result	method of calibration	95%CI	statistics	P price
	Min - Max	27.00 - 142.00	23.00 - 150.00				
	Missing	0	16				
Visit 3_ Creatinine CREA	Control group (N=149)			Wilcoxon signed rank test	(-3.65 , 0.4)	T = 2086	0.097
	Mean (SD)	72.09 (19.99)	74.57 (24.48)				
	Median (Q1, Q3)	68.00 (58.00, 83.40)	72.00 (59.68, 84.00)				
	Min - Max	37.60 - 174.00	42.80 - 240.00				
	Missing	2	39				
	Test group (N=151)			Wilcoxon signed rank test	(-1.3 , 2.3)	T = 3663.5	0.565
	Mean (SD)	71.97 (17.46)	72.12 (16.44)				
	Median (Q1, Q3)	70.00 (61.00, 79.00)	70.00 (61.83, 78.85)				
	Min - Max	27.00 - 142.00	41.50 - 149.00				
	Missing	0	27				
Visit 4_ Creatinine CREA	Control group (N=149)			Wilcoxon signed rank test	(-3 , 0.5)	T = 3124	0.185
	Mean (SD)	72.09 (19.99)	74.50 (22.65)				

variable	group	base line	final result	method of calibration	95%CI	statistics	P price
	Median (Q1, Q3)	68.00 (58.00, 83.40)	72.90 (60.50, 86.00)				
	Min - Max	37.60 - 174.00	39.00 - 240.00				
	Missing	2	22				
	Test group (N=151)			Wilcoxon signed rank test	(-1.95 , 1.55)	T = 3771.5	0.918
	Mean (SD)	71.97 (17.46)	72.59 (16.98)				
	Median (Q1, Q3)	70.00 (61.00, 79.00)	71.00 (62.00, 81.00)				
	Min - Max	27.00 - 142.00	26.00 - 151.00				
	Missing	0	21				

4 Urinary routine

4.1, and the urinary protein

(1) Comparison between the groups of the visiting viewpoints

variable	Test group (N=151)	Control group (N=149)	Total (N=300)	method of calibrati on	95%CI	statistic s	P price
Visit 1_ Urinary protein				Wilcoxon rank sum test	(0 , 0)	T = 10199.00	0.165
-	132 (93.0%)	121 (88.3%)	253 (90.7%)				
±	7 (4.9%)	7 (5.1%)	14 (5.0%)				
+	3 (2.1%)	9 (6.6%)	12 (4.3%)				
Average rank	136.676	143.445	140				
Missing	9	12	21				
Visit 2_ Urinary protein				Wilcoxon rank sum test	(0 , 0)	T = 6648.50	0.841
-	109 (90.1%)	97 (89.0%)	206 (89.6%)				
±	5 (4.1%)	8 (7.3%)	13 (5.7%)				
+	7 (5.8%)	4 (3.7%)	11 (4.8%)				
Average rank	115.054	115.995	115.5				
Missing	30	40	70				
Visit 3_ Urinary protein				Wilcoxon rank sum test	(0 , 0)	T = 4936.50	0.323
-	93 (85.3%)	85 (89.5%)	178 (87.3%)				
±	6 (5.5%)	7 (7.4%)	13 (6.4%)				
+	10 (9.2%)	3 (3.2%)	13 (6.4%)				

variable	Test group (N=151)	Control group (N=149)	Total (N=300)	method of calibration	95%CI	statistics	P price
Average rank	104.711	99.963	102.5				
Missing	42	54	96				
Visit 4_ Urinary protein				Wilcoxon rank sum test	(0 , 0)	T = 5377.00	0.280
-	88 (83.0%)	95 (88.8%)	183 (85.9%)				
±	13 (12.3%)	5 (4.7%)	18 (8.5%)				
+	5 (4.7%)	7 (6.5%)	12 (5.6%)				
Average rank	109.774	104.252	107				
Missing	45	42	87				

(2) Baseline and each view group

variable name	group	variate-value	-_ final result	±_ final result	+_ final result	amount to	P price
Visit 2_ Urinary protein	1	-_ base line	101	4	3	108	0.221
		±_ base line	6	0	1	7	
		+_ base line	0	0	3	3	
		amount to	107	4	7	118	
	2	-_ base line	87	5	0	92	0.152

variable name	group	variate-value	-_ final result	±_ final result	+_ final result	amount to	P price
		±_ base line	2	2	1	5	
		+_ base line	4	1	3	8	
		amount to	93	8	4	105	
Visit 3_ Urinary protein	1	-_ base line	91	4	4	99	0.032
		±_ base line	1	1	3	5	
		+_ base line	0	0	3	3	
		amount to	92	5	10	107	
	2	-_ base line	72	5	1	78	0.286
		±_ base line	4	0	0	4	
		+_ base line	5	1	2	8	
		amount to	81	6	3	90	
Visit 4_ Urinary protein	1	-_ base line	85	10	4	99	0.025
		±_ base line	2	2	1	5	
		+_ base line	0	1	0	1	
		amount to	87	13	5	105	

variable name	group	variate-value	-_ final result	±_ final result	+_ final result	amount to	P price
	2	-_ base line	83	3	4	90	0.841
		±_ base line	5	0	1	6	
		+_ base line	4	2	2	8	
		amount to	92	5	7	104	

4.2 Urine glucose

(1) Comparison between the groups of the visiting viewpoints

variable	Test group (N=151)	Control group (N=149)	Total (N=300)	method of calibrati on	95%CI	statistic s	P price
Visit 1_, urinary glucose				Wilcoxon rank sum test	(0 , 0)	T = 9826.00	0.776
-	129 (90.8%)	123 (89.8%)	252 (90.3%)				
±	4 (2.8%)	5 (3.6%)	9 (3.2%)				
+	9 (6.3%)	9 (6.6%)	18 (6.5%)				
Average rank	139.303	140.723	140				
Missing	9	12	21				
Visit 2_, urinary glucose				Wilcoxon rank sum test	(0 , 0)	T = 6711.00	0.658
-	110 (90.9%)	97 (89.0%)	207 (90.0%)				
±	2 (1.7%)	4 (3.7%)	6 (2.6%)				
+	9 (7.4%)	8 (7.3%)	17 (7.4%)				
Average rank	114.537	116.569	115.5				
Missing	30	40	70				
Visit 3_, urinary glucose				Wilcoxon rank sum test	(0 , 0)	T = 5029.50	0.451
-	99 (90.8%)	89 (93.7%)	188 (92.2%)				
±	0 (0.0%)	0 (0.0%)	0 (0.0%)				
+	10 (9.2%)	6 (6.3%)	16 (7.8%)				

variable	Test group (N=151)	Control group (N=149)	Total (N=300)	method of calibration	95%CI	statistics	P price
Average rank	103.858	100.942	102.5				
Missing	42	54	96				
Visit 4_, urinary glucose				Wilcoxon rank sum test	(0 , 0)	T = 5877.00	0.405
-	96 (90.6%)	93 (86.9%)	189 (88.7%)				
±	2 (1.9%)	3 (2.8%)	5 (2.3%)				
+	8 (7.5%)	11 (10.3%)	19 (8.9%)				
Average rank	105.057	108.925	107				
Missing	45	42	87				

(2) Baseline and each view group

variable name	group	variate-value	-_ final result	±_ final result	+_ final result	amount to	P price
Visit 2_, urinary glucose	1	-_ base line	103	2	1	106	0.506
		±_ base line	2	0	2	4	
		+_ base line	2	0	6	8	
		amount to	107	2	9	118	
	2	-_ base line	90	2	2	94	> 0.999

variable name	group	variate-value	-_ final result	±_ final result	+_ final result	amount to	P price
		±_ base line	2	1	1	4	
		+_ base line	2	1	4	7	
		amount to	94	4	7	105	
Visit 3_, urinary glucose	1	-_ base line	93	0	5	98	0.232
		±_ base line	2	0	1	3	
		+_ base line	2	0	4	6	
		amount to	97	0	10	107	
	2	-_ base line	76	0	1	77	-
		±_ base line	4	0	0	4	
		+_ base line	4	0	5	9	
		amount to	84	0	6	90	
Visit 4_, urinary glucose	1	-_ base line	91	0	3	94	0.801
		±_ base line	1	1	1	3	
		+_ base line	3	1	4	8	
		amount to	95	2	8	105	

variable name	group	variate-value	-_ final result	±_ final result	+_ final result	amount to	P price
	2	-_ base line	86	1	5	92	0.761
		±_ base line	2	0	2	4	
		+_ base line	3	1	4	8	
		amount to	91	2	11	104	

4.3 urinary red blood cells

(1) Comparison between the groups of the visiting viewpoints

variable	Test group (N=151)	Control group (N=149)	Total (N=300)	method of calibration	95%CI	statistics	P price
Visit 1_, urinary red blood cells				Wilcoxon rank sum test	(0 , 0)	T = 9336.00	0.214
-	128 (90.1%)	129 (94.2%)	257 (92.1%)				
±	0 (0.0%)	0 (0.0%)	0 (0.0%)				
+	14 (9.9%)	8 (5.8%)	22 (7.9%)				
Average rank	142.754	137.146	140				
Missing	9	12	21				
Visit 2_, urinary red blood cells				Wilcoxon rank sum test	(0 , 0)	T = 6243.00	0.124
-	109 (90.1%)	104 (95.4%)	213 (92.6%)				
±	0 (0.0%)	0 (0.0%)	0 (0.0%)				
+	12 (9.9%)	5 (4.6%)	17 (7.4%)				
Average rank	118.405	112.275	115.5				
Missing	30	40	70				
Visit 3_, urinary red blood cells				Wilcoxon rank sum test	(0 , 0)	T = 5110.50	0.672
-	103 (94.5%)	91 (95.8%)	194 (95.1%)				
±	0 (0.0%)	0 (0.0%)	0 (0.0%)				
+	6 (5.5%)	4 (4.2%)	10 (4.9%)				

variable	Test group (N=151)	Control group (N=149)	Total (N=300)	method of calibration	95%CI	statistics	P price
Average rank	103.115	101.795	102.5				
Missing	42	54	96				
Visit 4_, urinary red blood cells				Wilcoxon rank sum test	(0 , 0)	T = 5666.50	0.985
-	97 (91.5%)	98 (91.6%)	195 (91.5%)				
±	0 (0.0%)	0 (0.0%)	0 (0.0%)				
+	9 (8.5%)	9 (8.4%)	18 (8.5%)				
Average rank	107.042	106.958	107				
Missing	45	42	87				

(2) Baseline and each view group

variable name	group	variate-value	-_ final result	±_ final result	+_ final result	amount to	P price
Visit 2_, urinary red blood cells	1	-_ base line	101	0	6	107	-
		±_ base line	0	0	0	0	
		+_ base line	5	0	6	11	
		amount to	106	0	12	118	
	2	-_ base line	97	0	2	99	-
		±_ base line	0	0	0	0	

variable name	group	variate-value	-_ final result	±_ final result	+_ final result	amount to	P price
		+_ base line	4	0	2	6	
		amount to	101	0	4	105	
Visit 3_, urinary red blood cells	1	-_ base line	94	0	1	95	-
		±_ base line	0	0	0	0	
		+_ base line	7	0	5	12	
		amount to	101	0	6	107	
	2	-_ base line	85	0	4	89	-
		±_ base line	0	0	0	0	
		+_ base line	1	0	0	1	
		amount to	86	0	4	90	
Visit 4_, urinary red blood cells	1	-_ base line	92	0	4	96	-
		±_ base line	0	0	0	0	
		+_ base line	5	0	4	9	
		amount to	97	0	8	105	
	2	-_ base line	92	0	5	97	-

variable name	group	variate-value	-_ final result	±_ final result	+_ final result	amount to	P price
		±_ base line	0	0	0	0	
		+_ base line	3	0	4	7	
		amount to	95	0	9	104	

4.4 Urinary leukocytes

(1) Comparison between the groups of the visiting viewpoints

variable	Test group (N=151)	Control group (N=149)	Total (N=300)	method of calibration	95%CI	statistics	P price
Visit 1_, urinary white blood cells				Wilcoxon rank sum test	(0 , 0)	T = 9353.50	0.340
-	121 (85.2%)	122 (89.1%)	243 (87.1%)				
±	0 (0.0%)	0 (0.0%)	0 (0.0%)				
+	21 (14.8%)	15 (10.9%)	36 (12.9%)				
Average rank	142.63	137.274	140				
Missing	9	12	21				
Visit 2_, urinary white blood cells				Wilcoxon rank sum test	(0 , 0)	T = 6230.50	0.229
-	101 (83.5%)	97 (89.0%)	198 (86.1%)				
±	0 (0.0%)	0 (0.0%)	0 (0.0%)				
+	20 (16.5%)	12 (11.0%)	32 (13.9%)				
Average rank	118.508	112.161	115.5				
Missing	30	40	70				
Visit 3_, urinary white blood cells				Wilcoxon rank sum test	(0 , 0)	T = 5077.00	0.599

variable	Test group (N=151)	Control group (N=149)	Total (N=300)	method of calibration	95%CI	statistics	P price
-	100 (91.7%)	89 (93.7%)	189 (92.6%)				
±	0 (0.0%)	0 (0.0%)	0 (0.0%)				
+	9 (8.3%)	6 (6.3%)	15 (7.4%)				
Average rank	103.422	101.442	102.5				
Missing	42	54	96				
Visit 4_, urinary white blood cells				Wilcoxon rank sum test	(0 , 0)	T = 5610.50	0.822
-	91 (85.8%)	93 (86.9%)	184 (86.4%)				
±	0 (0.0%)	0 (0.0%)	0 (0.0%)				
+	15 (14.2%)	14 (13.1%)	29 (13.6%)				
Average rank	107.571	106.435	107				
Missing	45	42	87				

(2) Baseline and each view group

variable name	group	variate-value	-_ final result	±_ final result	+_ final result	amount to	P price
Visit 2_, urinary white blood cells	1	-_ base line	91	0	10	101	-
		±_ base line	0	0	0	0	

variable name	group	variate-value	-_ final result	±_ final result	+_ final result	amount to	P price
		+_ base line	7	0	10	17	
		amount to	98	0	20	118	
	2	-_ base line	87	0	6	93	-
		±_ base line	0	0	0	0	
		+_ base line	6	0	6	12	
		amount to	93	0	12	105	
Visit 3_, urinary white blood cells	1	-_ base line	89	0	3	92	-
		±_ base line	0	0	0	0	
		+_ base line	9	0	6	15	
		amount to	98	0	9	107	
	2	-_ base line	76	0	3	79	-
		±_ base line	0	0	0	0	
		+_ base line	9	0	2	11	
		amount to	85	0	5	90	

variable name	group	variate-value	-_ final result	±_ final result	+_ final result	amount to	P price
Visit 4_, urinary white blood cells	1	-_ base line	81	0	9	90	-
		±_ base line	0	0	0	0	
		+_ base line	9	0	6	15	
		amount to	90	0	15	105	
	2	-_ base line	82	0	11	93	-
		±_ base line	0	0	0	0	
		+_ base line	8	0	3	11	
		amount to	90	0	14	104	
